# Ionic Liquid Lignosulfonate: Dispersant and Binder for Preparation of Biocomposite Materials

**DOI:** 10.1002/anie.201907385

**Published:** 2019-07-25

**Authors:** Ryan Guterman, Valerio Molinari, Elinor Josef

**Affiliations:** ^1^ Colloids Department Max Planck Institute of Colloids and Interfaces (MPIKG) Am Mühlenberg 1 14476 Potsdam Germany

**Keywords:** biomass, ionic liquids, lignin, materials science, polymers

## Abstract

Ionic liquid lignins are prepared from sodium lignosulfonate by a cation exchange reaction and display glass transition temperatures as low as −13 °C. Diethyleneglycol‐functionalized protic cations inhibit lignin aggregation to produce a free‐flowing “ionic liquid lignin”, despite it being a high‐molecular‐weight polyelectrolyte. Through this approach, the properties of both lignin and ionic liquids are combined to create a dispersant and binder for cellulose+gluten mixtures to produce small microphases. Biocomposite testing pieces are produced by hot‐pressing this mixture, yielding a material with fewer defects and improved toughness in comparison to other lignins. The use of unmodified lignosulfonate, acetylated lignosulfonate, or free ionic liquid for similar materials production yields poorer substances because of their inability to maximize interfacial contact and complexation with cellulose and proteins.

## Graphical abstract

**Hot off the press**: Lignosulfonate is functionalized to create an ionic liquid with a glass transition temperature (*T*_g_) of −13 °C. After mixing with cellulose+gluten and hot‐pressing, composite materials are produced and have fewer defects and improved toughness in comparison to other lignins.

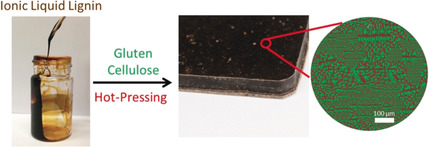

Lignin is the second most available biopolymer on earth with millions of tons produced every year as a byproduct of the pulp and paper industry.^[1]^ Its ready availability, high carbon content, and numerous reactive functional groups^[2]^ makes lignin a potentially useful carbon‐neutral polymer source for the fabrication of high‐value products.^[3]^ Despite these benefits, lignin extracted from plant matter possesses ill‐defined molecular structures whose properties, molecular weight, and chemical functionality are highly dependent on its isolation method and source,^[4]^ thus complicating their use. While some strategies seek to depolymerize lignin^[5]^ and forego any attempts to harness its polymeric structure, other promising approaches seek to utilize lignin as a functional additive^[6]^ to reduce the weight fraction of petroleum‐sourced polymers in materials, as a dispersant,^[7]^ or instead create wholly new materials primarily based on lignin.^[8]^ Their biodegradability makes them particularly suited towards these applications while the presence of both modified and unmodified hydroxy groups assist in dispersing a wide variety of substances, including dyes,^[9]^ coal slurries,^[10]^ cement,^[11]^ carbon nanotubes,^[12]^ and silica to prepare composite materials.^[13]^ In a review by Fatehi et al.,^[7]^ they show that different lignin structures, compositions, and modifications lead to either more specific or advantageous dispersing capabilities. Some common modifications include sulfomethlyation, hydroxyalkylation, oxidation, PEGylation (PEG=polyethylene glycol), and oxidation or ozone treatment to increase the presence of hydroxyl functionality. Ideally, a good lignin‐type dispersant should help to increase surface area of the dispersed components, produce smaller phases within the material, and improve performance for a given application. Despite these achievements, the supply of waste lignin from kraft and sulfite cooking is greater than its demand.^[6c]^ To more broadly incorporate lignin as a major component of composite materials and polymers,^[14]^ either chemical modification or new processing methods are necessary.^[3b]^ Chemical modifications by reaction at the alcohol groups^[15]^ are among the most common approaches and can improve miscibility with commodity polymers^[16]^ such as polypropylene and polystyrene. While these endeavors may reduce the petroleum weight‐fraction in the final material, complete replacement of synthetic polymers with biopolymers is most ideal. Cellulose is an excellent candidate for use in lignin composites thanks to its availability and high elastic modulus of the single fibers (values can reach above 100 GPa),^[17]^ and is commonly used in thermosetting composites,^[18]^ packaging,^[19]^ and extruded materials.^[20]^ Despite these developments, its use is limited by poor processability and low solubility. To facilitate better mixing of lignin and cellulose, new lignin‐based materials must be developed. One promising strategy utilizes ionic liquids (ILs) as solvents, which can dissolve large amounts of cellulose and aid in the fabrication processes. This was first shown by Rogers et al.,^[21]^ whose work has since motivated many research groups to explore new processing methods for fabricating cellulose‐based fibers, films, and organic/inorganic composites.^[22]^ Composite materials in particular harness strong interface interactions by hydrogen bonding between cellulose and other added components, such certain carbohydrates (starch, agarose, cyclodextrins), or proteins (keratin, wool, collagen), and leads to improved thermomechanical properties relative to just cellulose alone.^[23]^ Recently, ILs have been used for the fabrication of lignin‐cellulose composites with success.^[24]^ The IL here acts as a dispersant to allow lignin and cellulose to better interface with each other,^[25]^ and thus produce a more robust material. In these examples, the IL must be removed from the final product by solvent extraction, which significantly limits the dimensions of the produced materials and may compromise integrity. For example, fibers and films possess thin cross‐sections, which enable the removal of IL from the final material. Other items like tiles, panels, or casings do not have such thin cross‐sections and therefore cannot be fabricated in a similar fashion. Other techniques such as hot pressing or extrusion are often employed to produce materials with larger dimensions such as particle boards, foams, and molded composites. One solution is to functionalize lignin with ILs to promote better compatibility between lignin and cellulose during fabrication. Recently Gu and Bai et al. have shown that modification of the polyelectrolyte sodium lignosulfonate (**SLS**) with organic cations is feasible by cation exchange reactions,^[26]^ and is a simple approach that does not require utilization of the OH functionality. Ion‐exchange serves as a means to introduce functionality onto polyelectrolytes^[27]^ and often changes its electrical,^[28]^ chemical,^[29]^ and thermal properties.^[30]^ Also, it is a mild reaction tolerant to many functional groups. To date, this approach has not been examined as a viable way to alter either the chemical or physical properties of lignin or as a means to improve compatibilization with biopolymers.

In this context, we report a fabrication method using biopolymers, such as lignosulfonate, cellulose, and gluten, for producing panel composites. The modification of **SLS** with organic cations by cation exchange produces “ionic liquid‐lignin”, which acts as a dispersant and binder for plant‐based biopolymers and assists in the fabrication of lignin‐cellulose composites. Modification with different organic cations results in dramatic lowering of the glass‐transition temperature by hindering hydrogen‐bonding interactions between lignin macromolecules. We found that lignin modified with the tris‐[2‐(2‐methoxyethoxy)ethyl]amine (**TrisEG**; Figure 1) possessing an ethyleneglycol functionality effectively dispersed cellulose+gluten microphases (1–10 μm) while acting as a binder to improve mechanical properties. Composite materials were fabricated (small panels of 5×5×0.5 cm) with improved toughness (over 15 MJ m^−3^) over that of unmodified lignin (around 2 MJ m^−3^). This observation demonstrates the unique benefits of combing lignin with ionic liquids and further broadens the application of these waste materials.

**Figure 1:**
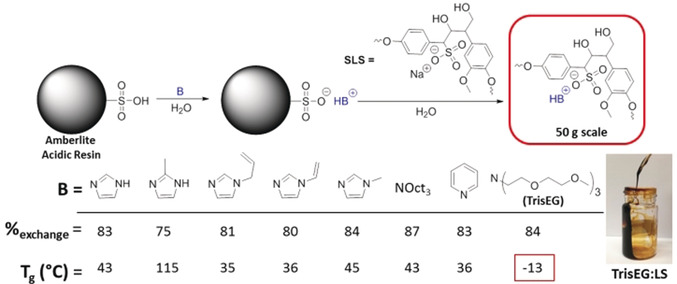
Top: Synthetic procedure for cation‐modified lignosulfonate. Bottom: The extent of cation modification was determined and the *T*_g_ value of the resulting materials measured. **TrisEG:LS** is a viscous liquid at room temperature.

We initially attempted cation exchange of **SLS** with different ammonium/imidazolium chloride salts to isolate the product by salt metathesis and separate the insoluble organic fraction. Instead we utilized a solid‐supported cation exchange resin to produce lignin with different organic cations (Figure 1; full description/discussion of the method can be found in on page S2 in the Supporting Information). We previously employed this methodology to prepare synthetic sulfonate polymers with ultra‐low *T*_g_ values^[31]^ and it has also been used elsewhere to prepare ionic liquids composed of amino acids.^[32]^ Dramatic changes in the *T*_g_ value were observed (see Figures S11–S18 in the Supporting Information), ranging from as low as −13 to 115 °C for the modified lignins, while no *T*_g_ was observed for **SLS**. In all cases, we observed the presence of the cation and the lignin protons in the ^1^H NMR spectrum, indicating that the exchange occurred and that no covalent bonds with lignin were formed/broken (see Figures S19–S26). The sodium content of the modified lignin was determined by inductively coupled plasma‐atomic emission spectroscopy (ICP‐AES), and showed a decrease in sodium content from 109 mg g^−1^ for **SLS** to approximately 5–15 mg g^−1^ in the exchanged product, providing strong evidence for the cation exchange reaction. Upon drying the product, a solid powder was isolated for all cation exchanged lignins except for **TrisEG:LS**, which was isolated as a highly viscous liquid (Figure 1, bottom). It was previously reported by us and others that polyelectrolytes containing the **TrisEG** structure have particularly low glass‐transition temperatures (*T*_g_; −57 °C) and possesses very high ionic conductivities.[^31^, ^33^] To date, these are among the very few examples of a free‐flowing polyelectrolyte and makes **TrisEG:LS** the first lignin‐based example with a *T*_g_ of −13 °C. Diethyleneglycol chains prevent aggregation of the anionic polyelectrolyte, resulting in a very low *T*_g_ value relative to conventional polyelectrolytes. Other organic cations have a diminished effect and produce lignins with a *T*_g_ value between 35–115 °C, while no *T*_g_ was observed for **SLS**. These results show that cation modification of **SLS** is a viable method to alter the thermal properties of lignin and introduce new functionality without hydroxy utilization.

The low *T*_g_ value and glycol functionality of **TrisEG:LS** may assist in increasing interfacial contact and facilitating better dispersion within lignin‐gluten composites,^[34]^ while also dissolving cellulose,^[35]^ making it a suitable candidate for composites. This finding is in addition to recent reports by Yoshizawa‐Fujita et al.,^[36]^ and Henderson et al.,^[37]^ who demonstrated the solubilizing properties of protic ionic liquids for cellulose and lignin, respectively. Particle board composites were prepared by hot pressing a wet mixture of different lignins/ionic liquid, gluten, and cellulose (Figure 2, top) following a procedure developed in our department. To determine the role of the lignin and IL in the composites, **SLS**, **TrisEG:LS**, the ionic liquid **TrisEG:MsOH**, or an acetylated version of **SLS** (**Ac:SLS**) was used within the trinary mixture. The preparation and testing consisted of a four‐step process (Figure 2, bottom; see page S3 for experimental details). Three series of four composites containing different amounts lignin/IL were prepared ranging from 6, 16, 27, and 38 wt % (see Tables S1 and S2). The appearance of the composites varied significantly with IL/lignin mixture and content. Scanning electron microscopy (SEM) imaging of the composite **4‐TrisEG:MsOH** revealed differentiated strands of cellulose fibers coated in IL (see Figure S1), and are even visible by optical microscopy (Figure 3 A). The well‐coated strands indicate favorable interfacial properties between cellulose and the IL, however their clear visibility at low magnifications indicates that the primary fibers, also known from paper, remain. In this case, **TrisEG** is a poor dispersant for cellulose and gluten and instead results in the persistence of micro‐ and even millimeter‐sized phases within the composite. Small‐angle X‐ray scattering (SAXS) was used to probe the nanostructure of the composites, with a detectable range of 1–60 nm. SAXS of **1‐**, **2‐**, **3‐**, and **4‐TrisEG:MsOH**, containing 6, 16, 27, and 38 wt % of TrisEG‐modified lignin, respectively, showed similar scattering down to *q*≈0.06 Å^−1^ (10 nm), where the plots being to diverge (see Figure S28 A). The change in the upturn at low *q* values indicates that **TrisEG: MsOH** changes either the structure or interaction with gluten or cellulose at larger length scales (the scattering from **TrisEG:MsOH** itself is negligible). Composites prepared with **SLS** appeared homogenous in composition with some slight cracking, however at higher **SLS** content (>27 wt %) phase separation was observed (see Figure S2). SEM analysis of **4‐SLS** showed microscale incorporation of cellulose into the matrix, although at higher magnifications fibers can be observed in some areas (see Figure S3). This stark contrast shows the ability for **SLS** to disperse cellulose/gluten and create smaller microphases. This dispersion is only possible up to a limit when particles of **SLS** separate and millimeter‐scale phases begin to appear (Figure 3 C). SAXS patterns of **1‐**,**2‐**, and **3‐SLS** are similar, indicating that the nanostructure of the polymer chains does not change upon increase of the **SLS** content (see Figure S28 B). However, a new nanoscale structure is revealed in **4‐SLS** and may suggest the appearance of a new nanophase. In agreement with the SEM analysis of **4‐SLS**, the new structure may indicate an upper limit for **SLS** as a dispersant. The critical role of hydroxy groups for assisting in dispersion is exemplified when they are acetylated prior to composite production. We found that replacing **SLS** with **Ac:SLS** yields highly phase‐separated composites on both the millimeter‐ and microscale (Figure 3 B; see Figure S4). A model diagram and optical images comparing the difference between **Ac:SLS** and **SLS** can be seen in Figures 3 B and 3 C. Very large domains up to 600 μm are observed for **Ac:SLS** and indicates poor interfacial stabilization. **SLS** appears to stabilize smaller microphases, however domains 50–200 μm in size are visible. Composites produced with **TrisEG:LS** displayed very little phase separation and no discernable domains on both the millimeter and microscale, even at higher loadings of 38 wt % (see Figure S5). Unlike **4‐SLS** and **4‐Ac:SLS**, no identifiable phases of lignin or cellulose down to about 10 μm could be observed in **4‐TrisEG:LS** and indicates excellent dispersion of lignin and cellulose (Figure 3 D; see Figures S6 and S7). SAXS measurements revealed that all composites produced from **TrisEG:LS** are similar (see Figure S28 C). Since the scattering of **TrisEG:LS** is negligible, only gluten and cellulose contribute to the scattering, and their structure at the 1–60 nm length scale appears to be almost unaffected by **TrisEG:LS** addition, which indicates that the dispersion abilities of **TrisEG:LS** are limited to the microscale.

**Figure 2:**
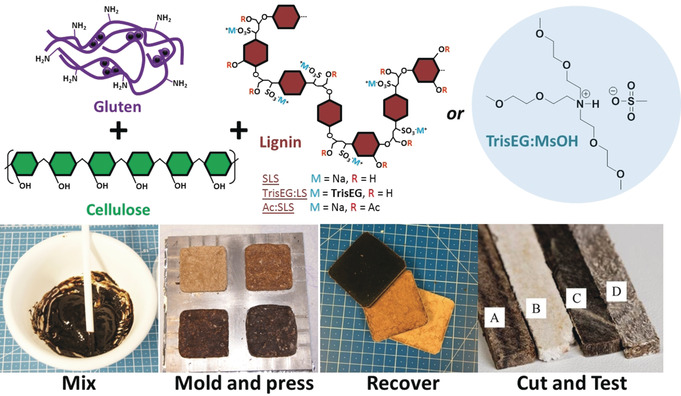
Top: All components used for preparing composite materials. Bottom: Stirring and molding (5×5×0.5 cm) of the gluten‐lignin‐cellulose mixture followed by hot‐pressing. After 1 hour the specimens are retrieved and cut for mechanical testing. Close up photo of 0.5 cm wide specimen strips prepared with **SLS** (A), **TrisEG:MsOH** (B), **TrisEG:LS** (C), and acetylated **AC:SLS** (D).

**Figure 3:**
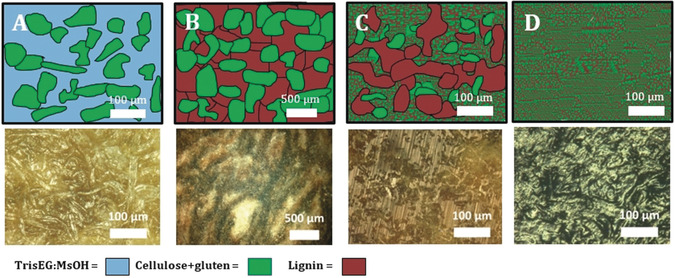
Model representation (top) and optical microscopy images (bottom) of composites A) **4‐TrisEG:MsOH**, B) **Ac:SLS** (38 wt %), C) **4‐SLS**, and D) **4‐TrisEG:LS**. The better dispersing abilities of **TrisEG:LS** promotes the formation of smaller microphrases and improved mechanical properties.

The combination of the “IL‐like” component and lignin macromolecular structure of **TrisEG:LS** improves interfacial contact and promotes the formation of small‐sized (ca. 5–10 μm) microphases. While **SLS** does act as a dispersant, it possesses an upper limit whereby millimeter‐sized phases begin to appear, and represents an upper limit for its incorporation and is undesirable for composite production. If the hydroxy groups are acetylated, the dispersion capabilities of lignin are completely eliminated and microphase interfaces are not stabilized, producing large millimeter‐sized phases. Unlike **TrisEG:MsOH** or the other lignins tested, **TrisEG:LS** represents a combination of properties that can be harnessed as a powerful dispersant to create small cellulose+gluten microphases even at very high loadings. The relationship between composite composition/structure and mechanical properties were then examined by bending and tensile tests. These results are summarized in Tables S1 and S2 with discussion on page S4. Briefly, **SLS** composites produced brittle materials while **TrisEG:MsOH** composites displayed the opposite trend. **TrisEG:LS** composites however were more tough and became more ductile while retaining toughness at higher lignin loadings. The overall superior properties of **TrisEG:LS** composites in comparison to **SLS** is in strong part due to the reduction of defects in the material. These observed results are in part reflected in the SAXS data, which in conjunction with optical and electron microscopy information, provides an explanation for these observed trends. SAXS of composites **1‐TrisEG**, **1‐SLS**, and **1‐TrisEG:LS** all look similar, and indicates similar nanoscale interactions in each specimen (see Figure S29 A). Given that the material is predominately cellulose and gluten and only 5 wt % of the third component, and that the scattering from **TrisEG:LS** and **TrisEG:MsOH** is negligible, the SAXS observed here is mostly a reflection of cellulose+gluten. The scattering of this composite does not equal the averaged scattering of individual components, indicating a change in the structure or interaction between gluten and cellulose following the processing (see Figure S30). It was not possible to process gluten and cellulose without addition of either **TrisEG: MsOH**, **SLS**, or **TrisEG:LS** and thus these controls were not examined. These specimens display very poor mechanical properties because of the formation of large, loosely connected phases that easily fracture. Moving to higher loadings, the SAXS pattern changes for the **SLS** and **TrisEG** series, while for **TrisEG:LS** there appears to be little change despite there being 38 wt % of the added lignin component, which itself exhibits negligible SAXS intensity. SAXS plots of **4‐TrisEG**, **4‐SLS**, and **4‐TrisEG:LS** are significantly different from one another (see Figure S29 B), indicating that the nanostructure of the specimen depends on the type of additive at a high concentration. For both **SLS** and **TrisEG:LS** an improvement in mechanical properties is observed at higher loadings, while for **TrisEG:MsOH** the material becomes worse. The dispersing abilities of both lignins assist in producing smaller microphases, and lead to better mechanical properties. Despite this, a compatibility limit is reached for **SLS**, as seen by phase separation optically and the change in SAXS pattern, leading to decreased toughness and breakage at low deformation (see Table S1). The appearance of no new nanostructure or interactions in the SAXS for the **TrisEG:LS** series indicates that similar cellulose+gluten interactions exist at every **TrisEG:LS** loading, however the homogenous nature of the composite optically and in the SEM images indicates that the cellulose+gluten phase becomes better dispersed with greater **TrisEG:LS** amount. Small microphases results in better mechanical properties by reducing the presence of defects (i.e. lignin agglomerates) which would behave as rupture points. The lignin agglomerates influence the brittleness of the material for samples prepared with **SLS** and have a lower deformation at break with increasing content of the lignin added to the composition. The opposite behavior is observed for **3‐TrisEG:LS** and **4‐TrisEG:LS**, where the composite becomes more ductile with the increase of lignin additive as a result of the homogeneous dispersion of the components and little to no agglomerates of lignin. Using **TrisEG:LS** allowed the preparation of fiberboard with tunable characteristics. Loadings up to 27 wt % produced homogeneous materials of high elastic modulus that can sustain very high forces, whereas a higher content (38 wt %) yielded a tough and ductile material of much higher deformation at break compared to untreated LS based composites for both fluxural and tensile tests (see Table S1 and S2), and also much higher maximum stress and toughness. These features are only observed in **TrisEG:LS** composites as conventional lignin‐based materials are commonly very brittle and break easily at low deformation, and demonstrate the benefits of IL‐lignin as a compatibilizer. Such **TrisEG:LS** specimens could withstand 100 cycles of stress from 0.1 to 9 MPa without breaking (see Figure S8). Composites containing this loading of **TrisEG:LS** still possess good mechanical properties, however the cellulose+gluten microphases are separated by a “sea” of free **TrisEG:LS**, thus resulting in a more ductile material. This wood‐rubber‐like material of a high toughness could be used for applications in which the material requires to be slightly bent or folded without breaking, applications usually covered by polymers or resins. Comparing the mechanical properties of produced composites to medium density fiberboard (MDF) provides a suitable comparison (Figure 4 B). MDF materials are cheap particle boards held together with the help of adhesives or resins, and often exhibit decent mechanical properties with low toughness because of their ease to break (Figure 4 B). Similar to MDF, one of the strengths of the here‐presented composite is the use of readily found or produced materials, but the here presented materials have a higher stiffness, achieved by using **SLS** (Figure 4 B, yellow line) or **TrisEG: MsOH** (Figure 4 B, green line), or a high toughness, unconventional for lignin based materials, by employing the novel **TrisEG:LS** at high concentration (Figure 4 B, red line). These materials have the great advantage of being formaldehyde‐ and phenol‐free, with the benefit of being manufactured with the same technology (hot pressing) used nowadays for the preparation of particle boards.

**Figure 4:**
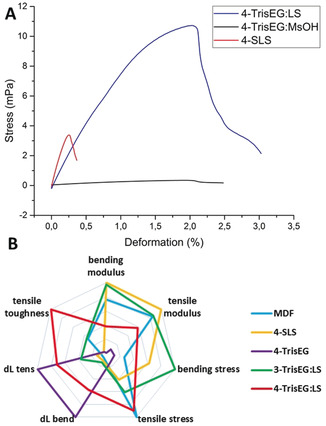
A) Stress‐strain curve of the tensile tests for composites containing **4‐TrisEG:LS**, **4‐TrisEG:MsOH**, and **4‐SLS** (38 wt % lignin/IL). B) Radar plot comparing medium density fiberboard (MDF) to the prepared composites.

In conclusion, cation exchange is an effective and simple method to modify the thermophysical properties of waste **SLS**. Sodium was replaced with eight different organic cations using a cation exchange resin and resulted in a dramatic decrease in the *T*_g_ value of lignin. The **TrisEG** cation however was found to separate lignin macromolecules to produce a flowable ionic liquid lignin with a *T*_g_ of −13 °C. This phenomenon is a result of the flexible diethyleneglycol chains on the nitrogen atom, and dramatically improves the mobility of lignin. Composites containing a mixture of **TrisEG:LS**, cellulose, and gluten as a model elastic polymer were prepared by hot‐pressing and different microphases were observed depending on the mixture. We show that only the combined properties of IL and lignosulfonate in one molecule can disperse cellulose and gluten at high concentrations to create cellulose and gluten microphases of less than 10 μm in diameter without lignin phase separation. This feature translates to better mechanical properties in comparison to other lignins tested. The retention of OH functionality and low *T*_g_ value of the modified lignin is essential to fabricate tough composites that are resistant to high stress. This aspect makes cation exchange a particularly attractive approach for introducing new functionalities while preserving the essential properties of lignin. We believe the introduction of cations containing task‐specific functions can further broaden the utility of this method and help to increase of the value of waste lignin.

## Conflict of interest

The authors declare no conflict of interest.

## Supporting information

As a service to our authors and readers, this journal provides supporting information supplied by the authors. Such materials are peer reviewed and may be re‐organized for online delivery, but are not copy‐edited or typeset. Technical support issues arising from supporting information (other than missing files) should be addressed to the authors.

Supplementary
