# Supplementary material for: Ionic Liquid Lignosulfonate: Dispersant and Binder for Preparation of Biocomposite Materials
Source: Angew Chem Int Ed Engl. 2019 Jul 25;58(37):13044–50. doi: 10.1002/anie.201907385 (PMC7687102; doi:10.1002/anie.201907385)
Supplement: Supplementary file 1 — Supplementary [file ANIE-58-13044-s001.pdf]

Angewandte Chemie
Eine Zeitschrift der Gesellschaft Deutscher Chemiker

Supporting Information

Ionic Liquid Lignosulfonate: Dispersant and Binder for Preparation of Biocomposite Materials

Ryan Guterman, Valerio Molinari,* and Elinor Josef*

anie_201907385_sm_miscellaneous_information.pdf

Ionic Liquid Lignosulfonate as a Dispersant and Binder for the preparation of Biocomposite Materials

Ryan Guterman*, Valerio Molinari*, Elinor Josef

Max Planck Institute of Colloids and Interfaces (MPIKG), Colloids Department, Am Mühlenberg 1, 14476 Potsdam, Germany.

Table of Contents

Experimental methods and Materials……………………………………………………………………………………………**2**

Composite composition and mechanical testing data……………………….……………………………………………**4**

Scanning electron microscopy (SEM) and optical microscopy images………………………………………….….**6**

Stress-strain curves………………………………………..……………………………………………………………………….**9**

Differential Scanning Calorimetry (DSC) thermographs……………………………..…………………………..……**10**

^{1}H Nuclear Magnetic Resonance spectra……………………………………………………...……….…………….**15**

^{13}C{^{1}H} Nuclear Magnetic Resonance spectra………..…………………………..……………………..………………**20**

Small Angle X-ray Scattering (SAXS)………………………..…………………………..………………………..………………**21**

Materials

Sodium lignosulfonate (**SLS**; 93%) was purchased from Roth and used as received. Tris-[2-(2-methoxyethoxy)ethyl]amine (**TrisEG**; 94%) was purchased from Merck and used as received. Imidazole (≥99 %), 1-vinylimidazole (≥99 %), trioctylamine (98 %), pyridine (99.8 %), gluten from wheat, and cellulose fibers (medium) were purchased from Sigma Aldrich and used as received. Amberlite® IR120 was purchased from Sigma Aldrich and rinsed with deionized water prior to use. ^{1}H and $^{13}C\{^{1}H\}$-NMR spectra were collected on a Bruker DPX-400 spectrometer. Thermogravimetric analysis (TGA) experiments were conducted using a Netzsch TG209-F1 Libra. An aluminum crucible was used for the measurement of ~10 mg of sample under flow of nitrogen (10 mL/min). The samples were heated at a rate of 2.5 K/min to 600 °C. Differential scanning calorimetry (DSC) experiments were performed on a PerkinElmer DSC-1 instrument at a heating/cooling rate of 10 K/min under nitrogen flow and cycled five times. Glass transition temperatures (T_g) was acquired by determining the temperature on the thermal curve corresponding to half the heat flow differences between the extrapolates onset and extrapolated end. This was performed using Netzsch Proteus Thermal Anlaysis software. Melting points were acquired from the final heating cycle. Inductively coupled plasma optical emission spectrometry (ICP-OES) was performed on a Perkin Elmer Optima 8000. Infrared spectroscopy was conducted on a Nicolet iS 5 FT-IR spectrometer.

Preparation of cation-exchanged lignosulfonate

Two different procedures were employed depending on the amount of desired material. To prepare cation-exchanged lignosulfonates on the order of 1-2 g, Amberlite® IR120 (5.00 g) was added to a 10 mL vial and rinsed with deionized water (3 x 5 mL) followed by the addition of fresh water (6 mL) and base (11.75 mmol; Imidazole, 0.80 g; 2-methylimidazole, 0.96 g; 1-allylimidazole, 1.27 g; 1-vinylimidazole, 1.10 g, 1-methylimidazole, 0.96 g; pyridine, 0.92 g; **TrisEG**, 3.79 g.). The vials were shaken overnight using a wrist-action shaker before being rinsed by water, ethanol, acetone, and then water on a fritted filter to remove excess base. The resin was then added to a **SLS** solution (0.40 g, 5 mL H_2O) and shaken overnight using a wrist-action shaker. The resin was then separated from solution by gravity filtration and the water evaporated *in vacuo* with heating leaving a dark power in quantitative yields. The isolated material when **TrisEG** was used was a dark viscous oil.

Preparation of **TrisEG**:**LS** on a larger scale was performed using a column. Amberlite® IR120 (157.43 g) was added to a column (3 cm diameter) fitted with a stopcock and rinsed with deionized water (3 L). **TrisEG** (119.35 g, 367.88 mmol) was then dissolved in 1 L deionized water and passed through the column slowly. The resin was then rinsed with water (3 L) to remove excess **TrisEG**. Then 85.69 g **SLS** was dissolved in water (500 mL) and slowly passed through the column and the cation-exchanged lignin isolated in a 1 L beaker. Volatiles were evaporated by heating in an oven at 90 °C for 12 hours which removed most of the water. More complete drying was achieved by heating the oil at 90 °C *in vacuo* for 24 hours, which resulted in a material with ~1 wt% H_2O content as determined by KF-titration.

Method discussion

Salt-metathesis is routinely performed to synthesize a variety of ILs and is high yielding when the organic salt is sufficiently insoluble and precipitates from solution. In this case however the exchanged product was too soluble in water and no precipitate was formed. To achieve the cation exchange and isolation of the lignin product, we employed the use of a resin. A strong acidic resin (Amberlite® IR120; sulfonic acid functionalized) was treated with different nitrogen bases (Figure 1) to form the protic salt, then cation exchanged with **SLS**. The spent resin was separated by gravity filtration and water evaporated to isolate the cation-modified lignin in quantitative yields. The resin was regenerated with a strong acid and reused multiple times. This methodology is simple, amenable to larger scale production, and requires no heating or toxic reagents in order to modify the thermal

and chemical properties of lignosulfonates. Unlike salt metathesis, where the precipitation of one product helps to drive the reaction forwards, the exchange of cations between two identical anions and no precipitation leads to incomplete conversion; however the use of excess ammonium-functionalized resin can favour high exchange yields to produce the desired product.

The T_g range from as low as -13 °C to 115 °C for the modified lignins and the absence of an obervable T_g for **SLS** indicates a change in the intermolecular forces between the lignin macromolecules as a result of the organic cation and that the cation structure is significant. While some sodium ions remained in the product after exchange, ion-exchange reactions with macromolecules are usually less complete in comparison to small molecules. In this case the vast majority (>80%) of sodium ions were exchanged with different organic cations and removed from solution using the exchange resin.

Preparation of the biocomposite

Water (17.0 mL) was added to the desired amount of lignin and stirred until complete dissolution. Gluten (6.0 g) and cellulose (10.0 g) were then mixed together as powders, either with the help of a rod or a mixer, until the powders obtained a homogeneous colouration. The lignin solution was poured into the gluten-cellulose powder mixture and the resulting dough kneaded by hand or with a mixer until all the liquid was absorbed by the powder. The wet dough was then placed into a custom made aluminum mold (5x5x0.5 cm) and placed into a hot-press previously heated to 130°C. The pressing of the specimen was performed gradually (every 5 minutes) to allow a gentle removal of the water, until the pressure of 10 bar was recorded on the instrument. Once the final pressure was reached, the specimens were cooled to room temperature under pressure and then recovered.

Sample Preparation: A band saw was used to cut the specimen in different thickness for flexural (0,5 cm thick) and tensile tests (0,2 cm thick) .

Mechanical Testing

All mechanical tests were measured using a Zwick mechanical tester zwickiLine Z2.5 equipped with a loadcell of 1 kN. Elastic modulus was manually calculated at 0,05–0,25% of strain.

All the measurements were recorded using the software TestXpert II V3.71.

Flexural test: A three-point-bending test was performed with a 2.5 kN mechanical testing machine (zwicki, Zwick Roell), equipped with a 1 kN load cell. The strains were measured by cross-head travel. The samples were loaded with a gap of 3 cm, a maximum force of 800 N, a preload of 0,5 N and a test speed of 0,025 mm s^{-1} .

Tensile test: Tensile test was performed with a 2.5 kN mechanical testing machine (zwicki, Zwick Roell), equipped with a 1 kN load cell. The strains were measured by cross-head travel. The samples were loaded with a gap of 3 cm, a maximum force of 800 N, a preload of 1 N and a test speed of 0,02 mm s^{-1} .

Cyclic test: The cycling test was performed until 9MPa of maximum stress and recovered until 1 N (on specimens of 30 mm) before the stress was removed to restart the cycle (100 times).

Small angle X-ray scattering (SAXS)

SAXS was carried using a Nanostar (Bruker AXS, Karlsruhe, Germany) device, equipped with a SIEMENS KFF CU 2K-90 X-ray tube, operating at 40 kV and 100 mA and generating an X-ray beam with a wavelength of 1.5418 Å (Cu Kα radiation) and a focal spot size of 550 µm. Silver behenate standard was used to calculate beam center and the exact sample-to-detector distance. 2D scattering plots were corrected for background and transmission.

TableS1: Results of 3 point bending test of specimens prepared with **SLS**, **TrisEG:LS**, and **TrisEG:MsOH**.

Sample	Lignin/IL	Content (wt%)	Emod (MPa)	Max Stress (MPa)	dL at break (%)	Flexural toughness (MJ/m^3)
**1-SLS**	**SLS**	6	67,6	1,38	2,34	1,08
**2-SLS**		16	144,75	1,92	1,57	1,81
**3-SLS**		27	235	2,47	1,39	1,99
**4-SLS**		38	248	2,39	1,27	1,86
**1-TrisEG:LS**	**TrisEG:LS**	6	82	1,55	2,35	2,12
**2-TrisEG:LS**		16	107	1,74	2,59	2,70
**3-TrisEG:LS**		27	244	3,81	3,17	6,71
**4-TrisEG:LS**		38	96	1,68	12,63	15,28
**1-TrisEG:MsOH**	**TrisEG:MsOH**	6	8,775	1,06	7,68	6,56
**2-TrisEG:MsOH**		16	18,2	0,87	7,99	3,62
**3-TrisEG:MsOH**		27	1,96	0,45	22,1	4,61
**4-TrisEG:MsOH**		38	0,86	0,07	24,1	0,55

TableS2: Results of tensile test of specimens prepared with **SLS**, **TrisEG:LS**, and **TrisEG:MsOH**.

Sample	Lignin/IL	Content (wt%)	Emod (MPa)	Max Stress (MPa)	dL at break (%)	Toughness (MJ/m^3)
**1-SLS**	**SLS**	6	664	0,45	0,401	0,13
**2-SLS**		16	1049	8,2	1,02	4,9
**3-SLS**		27	1784	4,96	0,41	1,2
**4-SLS**		38	1812	3,47	0,28	0,57
**1-TrisEG:LS**	**TrisEG:LS**	6	1132	1,94	0,37	0,47
**2-TrisEG:LS**		16	1920	6,21	0,48	1,82
**3-TrisEG:LS**		27	1525	7,36	1,10	5,35
**4-TrisEG:LS**		38	1023	10,78	2,14	15,33
**1-TrisEG:MsOH**	**TrisEG:MsOH**	6	-	-	-	-
**2-TrisEG:MsOH**		16	864	1,91	1,27	1,3
**3-TrisEG:MsOH**		27	157	1,66	2,99	2,4
**4-TrisEG:MsOH**		38	26	0,31	2,13	0,48

Discussion of the Bending and Tensile Tests

Composites prepared using **SLS** exhibited mechanical properties typical of brittle materials, where the bending modulus increased with increasing **SLS** content up to 27 wt% (Table S1, **3-SLS**), but also broke at low deformation (<2%). This brittleness is likely a result of the defects caused by phase-separated lignin which causes points of rupture in the material. Specimens prepared using **TrisEG:MsOH** displayed the opposite effect, with a progressively decreasing elastic modulus and rupture at 24 % of deformation in bending tests. (Table S1, entry **4-TrisEG:MsOH**) This can be ascribed to the lack of cohesion between grains and the presence of **TrisEG:MsOH** which simply fills the the composite's free-volume rather than dispersing and binding cellulose+gluten phases.

Composites containing **TrisEG:LS** were much more stiff to initial deformation in comparison, but also exhibited a higher deformaton at break at 38 wt% content (~13% for **4-TrisEG:LS** compared to ~1% for **4-SLS**). The elastic modulus is comparable to the **SLS** samples, but a higher maximum stress was measured when 27 wt% **TrisEG:LS** was employed (**3-TrisEG:LS** vs **3-SLS**). We attribute this difference to the fewer defects in the specimen. The plasticizing effect becomes dominant at 38 wt% **TrisEG:LS** where the stress-strain curve appears more similar to a ductile material rather than a typical particle board with a decrease of both Emod and stress maximum, and a dL jump from 3 to 12.6% yielding the toughest material of the materials here reported (~15 MJ/m^3). Tensile tests displayed a similar trend to the 3 point bending tests (Table S2). An increase in elastic modulus from 664 to 1812 MPa was observed for **SLS** samples with greater lignin content and a low deformation at break of <1 %. Composite **1-TrisEG:LS** again shows that **TrisEG:LS** is a good dispersant and binder for the preparation of such fiberboard, yielding materials with good resistance to initial deformation (elastic modulus of GPa range), high maximum stress (up to 10 MPa), and particularly high toughness in comparison to **SLS** containing samples (15 MJ/m^3, **4-TrisEG:LS** vs 0,57 MJ/m^3, (**4-SLS**). The much superior maximum stress for **4-TrisEG:LS** compared to **4-SLS** (~10 MPa vs ~3 MPa in Figure 4A) was achieved by the inhibition of defects in the composite, leading to a higher force required for the rupture of the specimens.

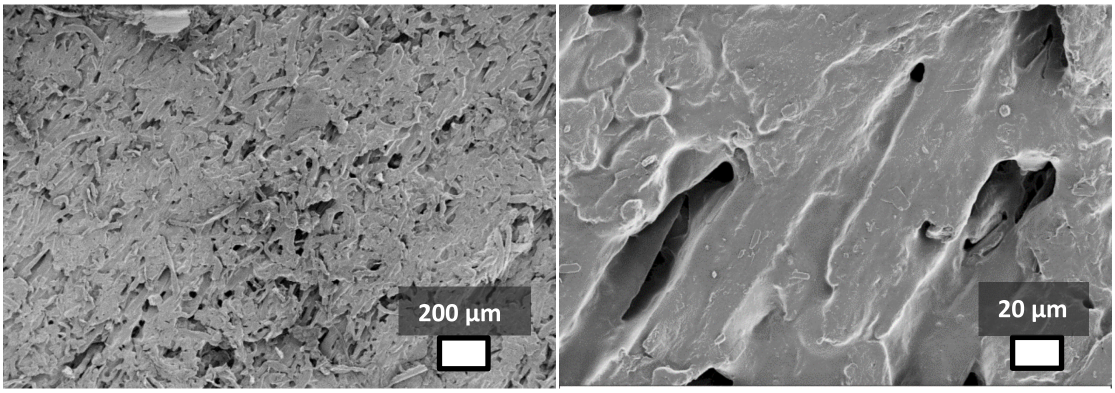

Figure S1. SEM images of composite **4-TrisEG:MsOH.**

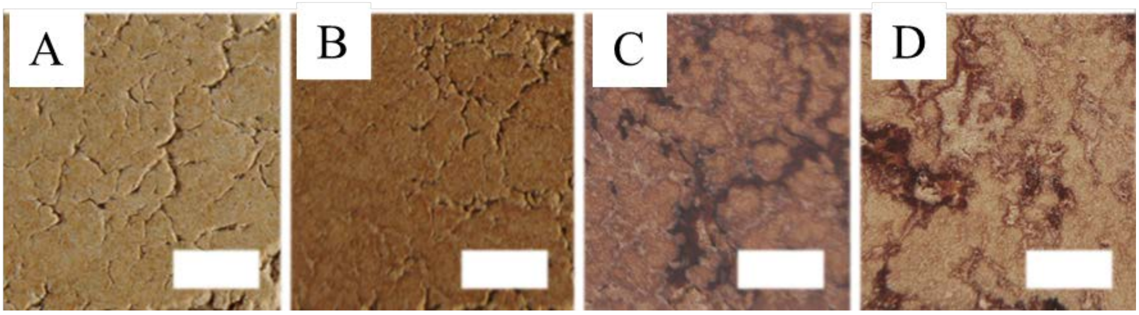

Figure S2. Optical microscopy images of A) **1-SLS**, B) **2-SLS**, C) **3-SLS**, and D) **4-SLS**. Scale bars are 1 cm long.

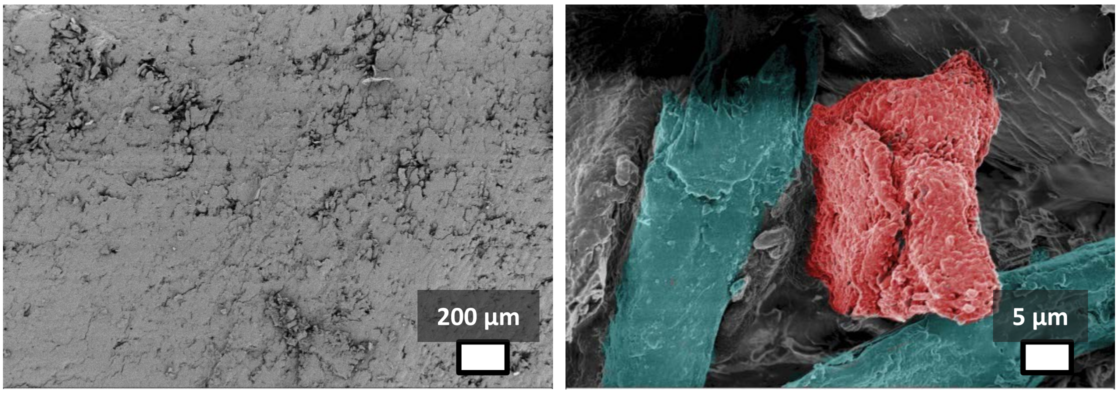

Figure S3. SEM images of composite **4-SLS**. Cellulose fibers (blue) and lignin particles (red) are embedded between mixed phases.

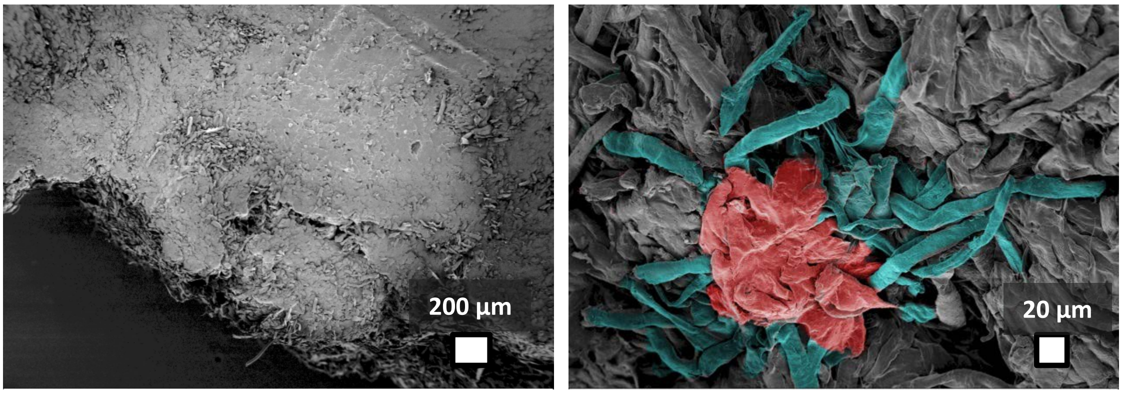

Figure S4. SEM images of composite **Ac:LS** (38 wt% acetylated lignin)**.** Cellulose fibers (blue) and lignin particles (red) are easily distinguishable and not mixed.

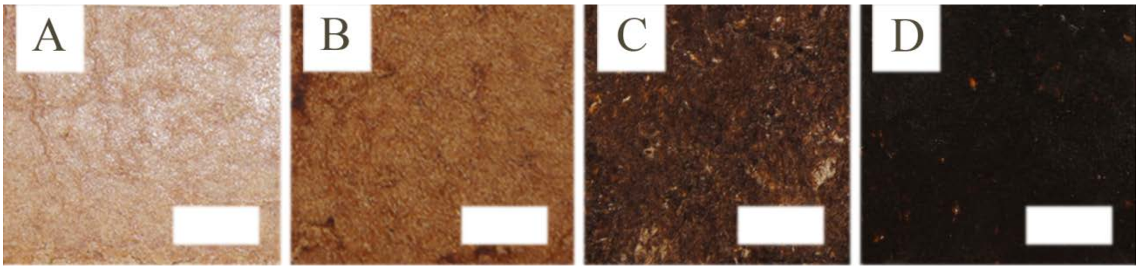

Figure S5. Optical microscopy images of A) **1-TrisEG:LS**, B) **2- TrisEG:LS**, C) **3- TrisEG:LS**, and D) **4-TrisEG:LS**. Scale bars are 1 cm long.

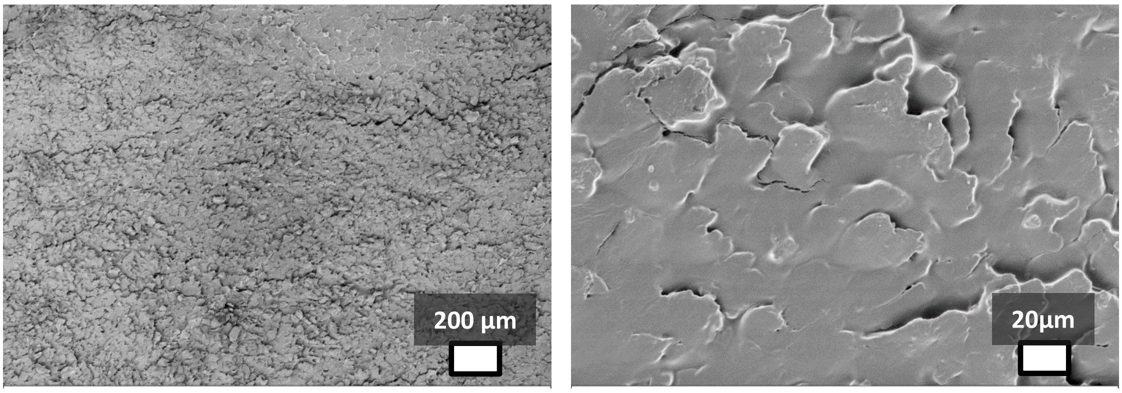

Figure S6. SEM images of composite **4-TrisEG:LS**. No cellulose or other distinct particles can be observed, however the mixed phases appear to be composed of different grains.

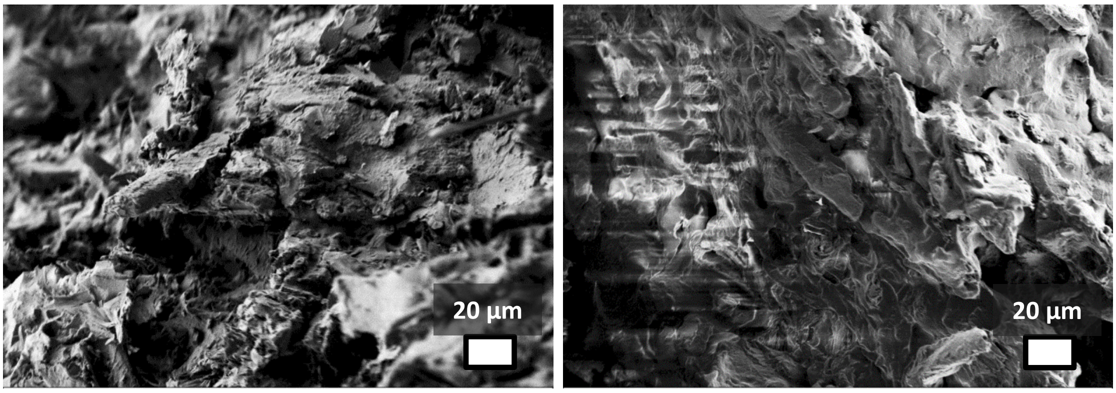

Figure S7. SEM images of a cross-section of composite **4-TrisEG:LS**. The interior is composed a mixed grains containing with no evidence of free cellulose fibers.

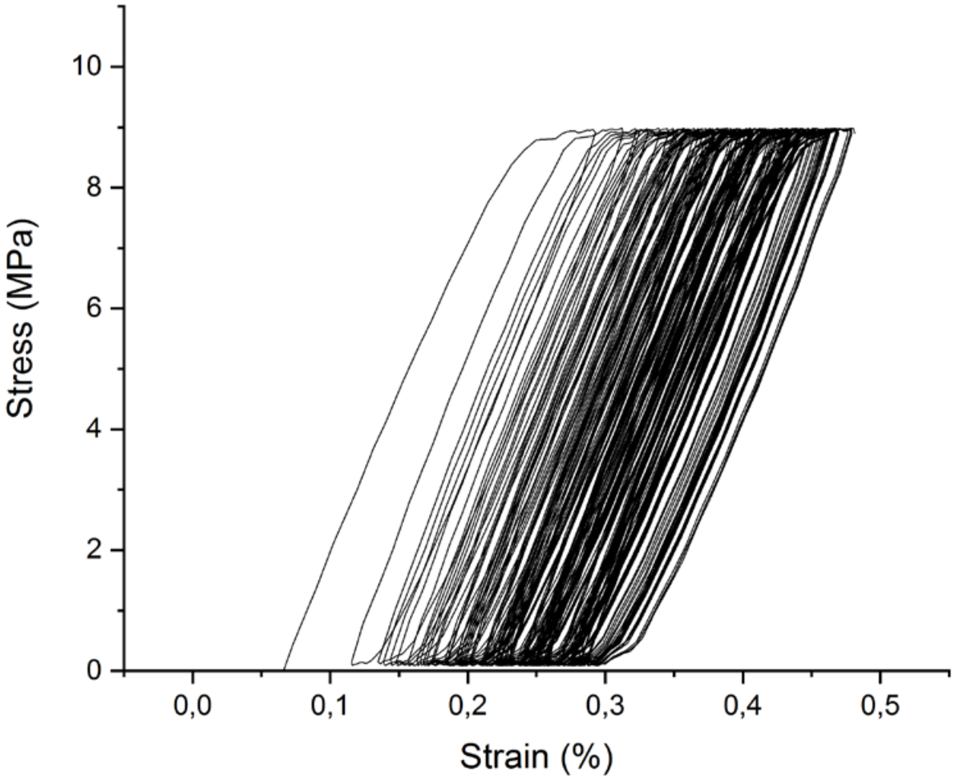

Figure S8. Cyclic tensile test of specimen prepared with **4-TrisEG:LS**.

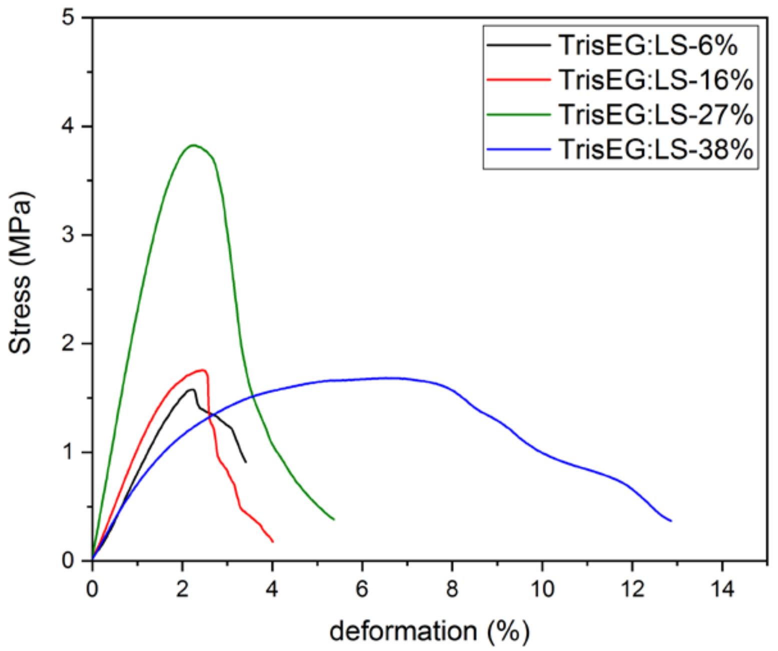

Figure S9 Bending test of specimens with different concentration of **TrisEG:LS** (from 6 to 38 wt%).

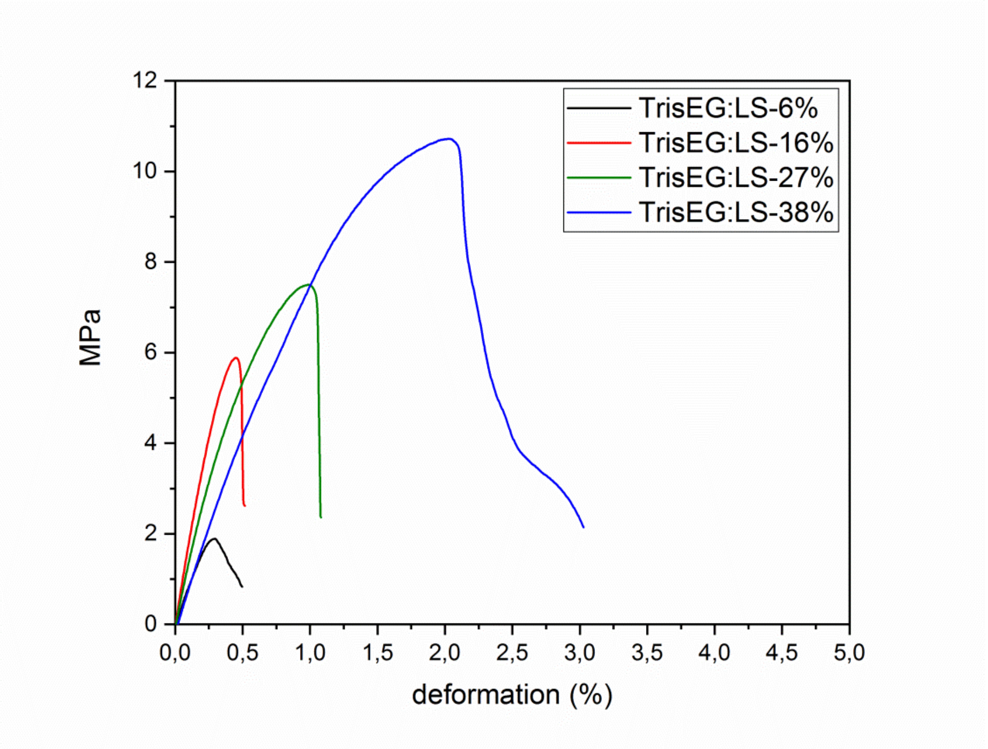

Figure S10. Tensile test of specimens with different concentration of **TrisEG:LS** (from 6 to 38 wt%).

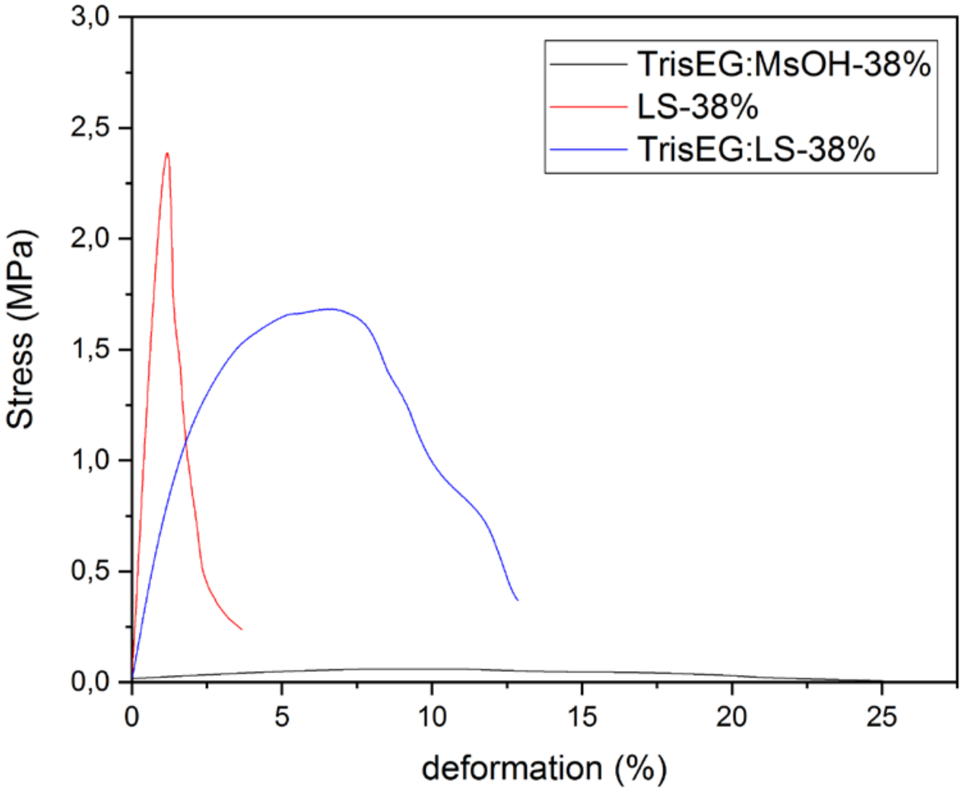

Figure S11. Bending test of specimens with different lignin types.

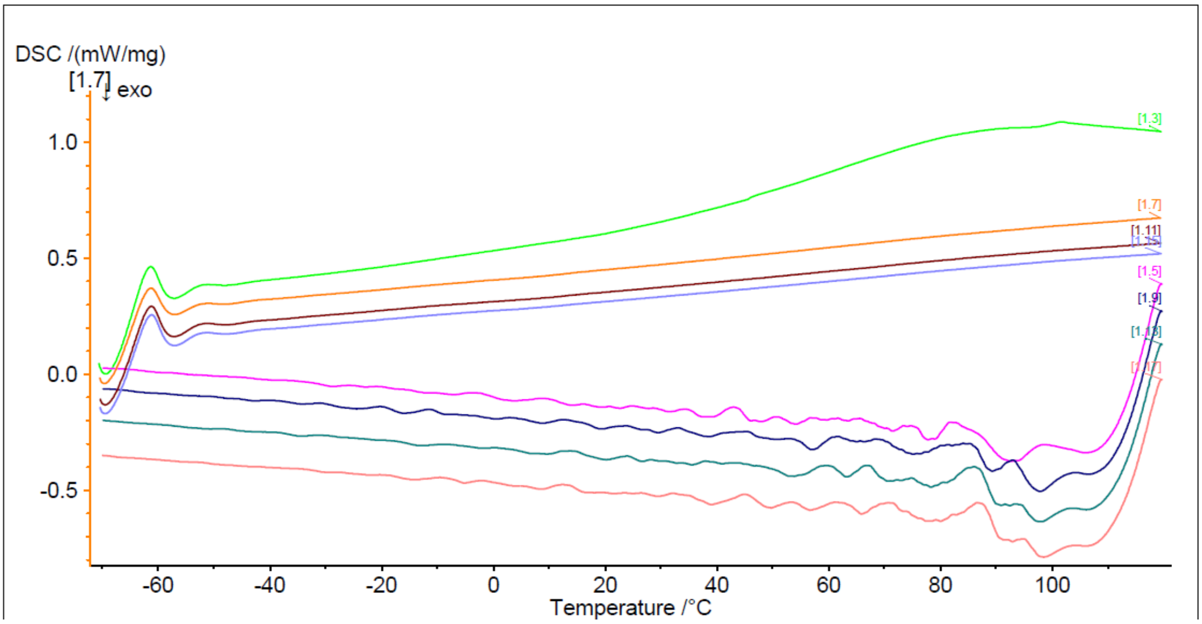

Figure S11. Differential scanning calorimetry of **4-TrisEG:LS** in nitrogen atmosphere, heating ramp of 10 K min^{-1}. The T_g of **TrisEG:LS** is not observed at -13 °C, indicating good mixing between cellulose/gluten with lignin. Plotted as "exo down".

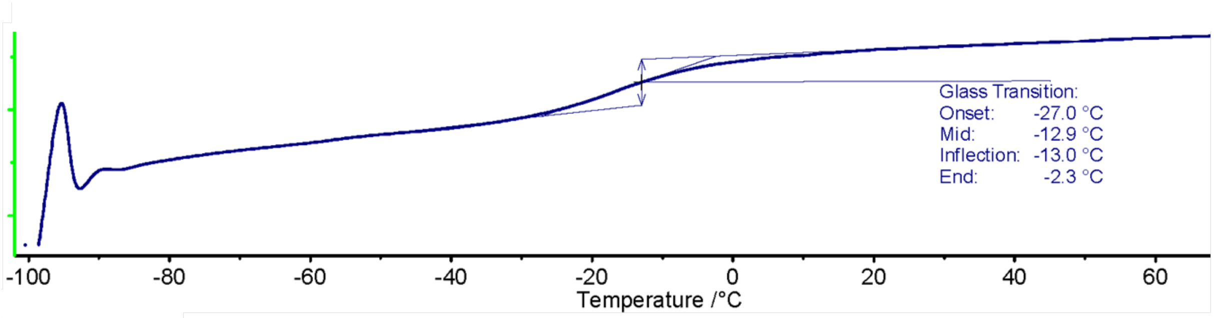

Figure S12. DSC of **TrisEG:LS**. Thermograph shown here acquired on the third heating. Plotted as "exo down".

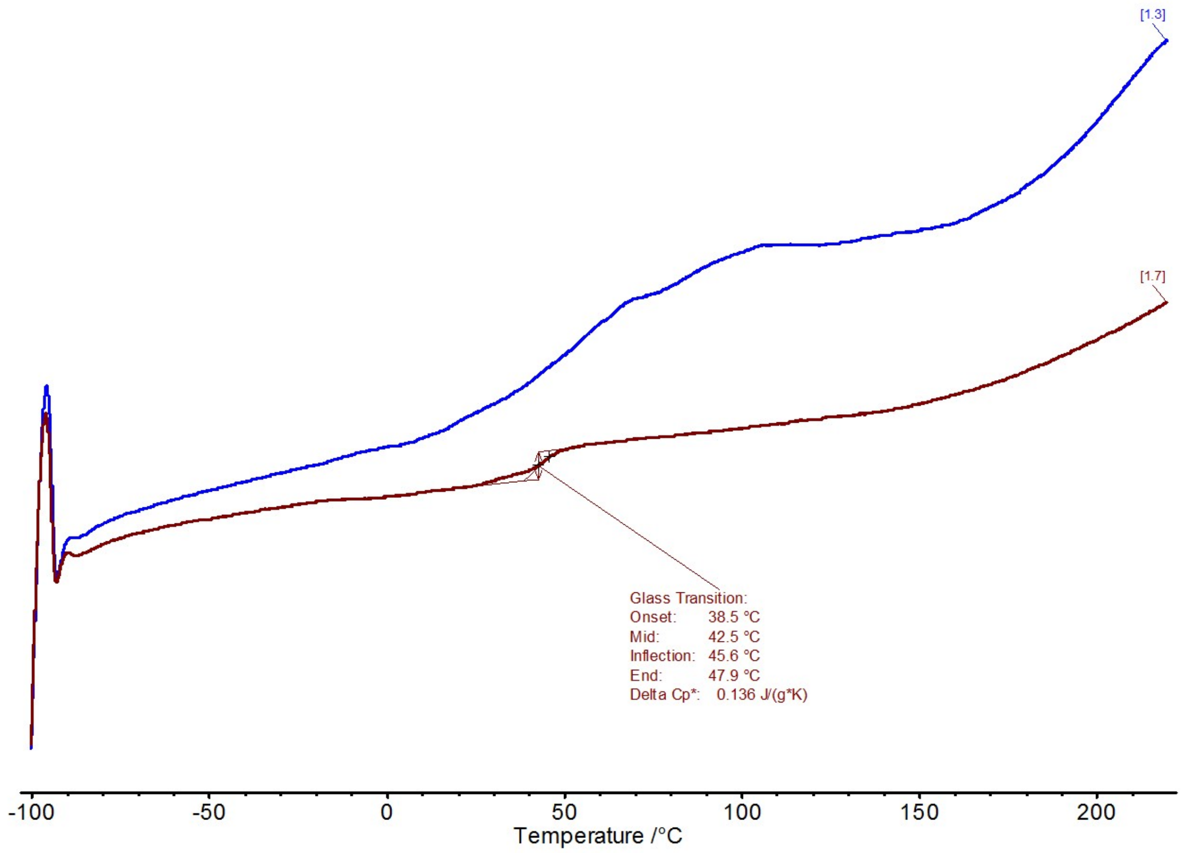

Figure S13. DSC of imidazolium functionalized LS. Traces 1.3 and 1.7 correspond to the second and third heatings. Plotted as "exo down".

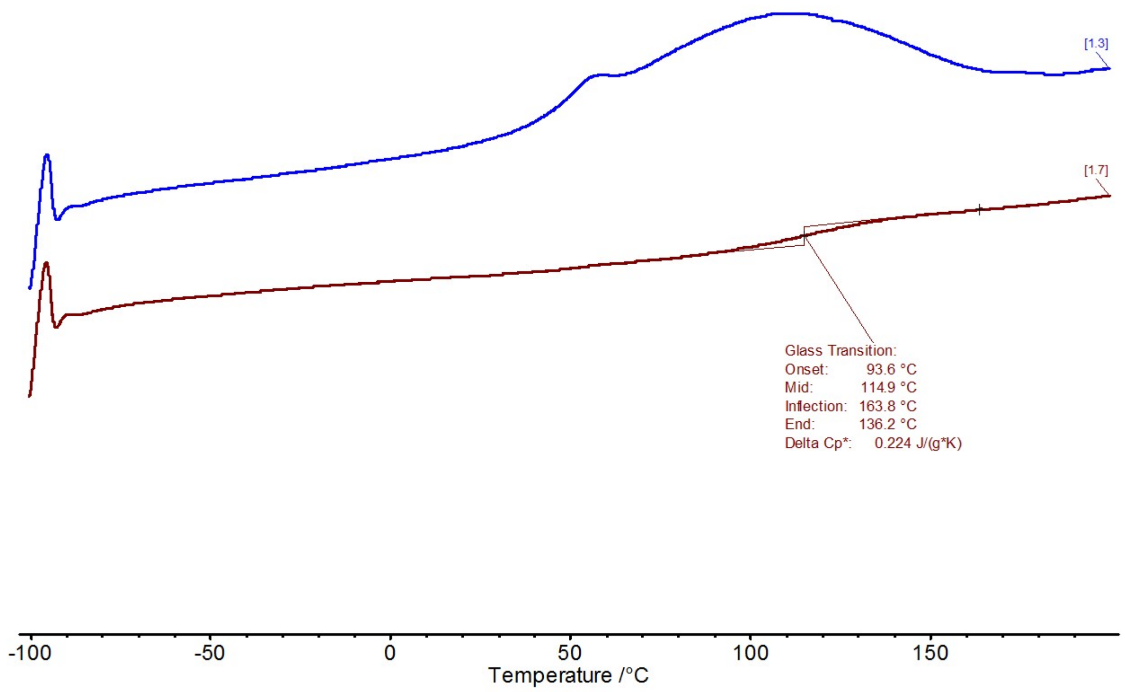

Figure S14. DSC of 2-methylimidazolium functionalized LS. Traces 1.3 and 1.7 correspond to the second and third heatings. Plotted as “exo down”.

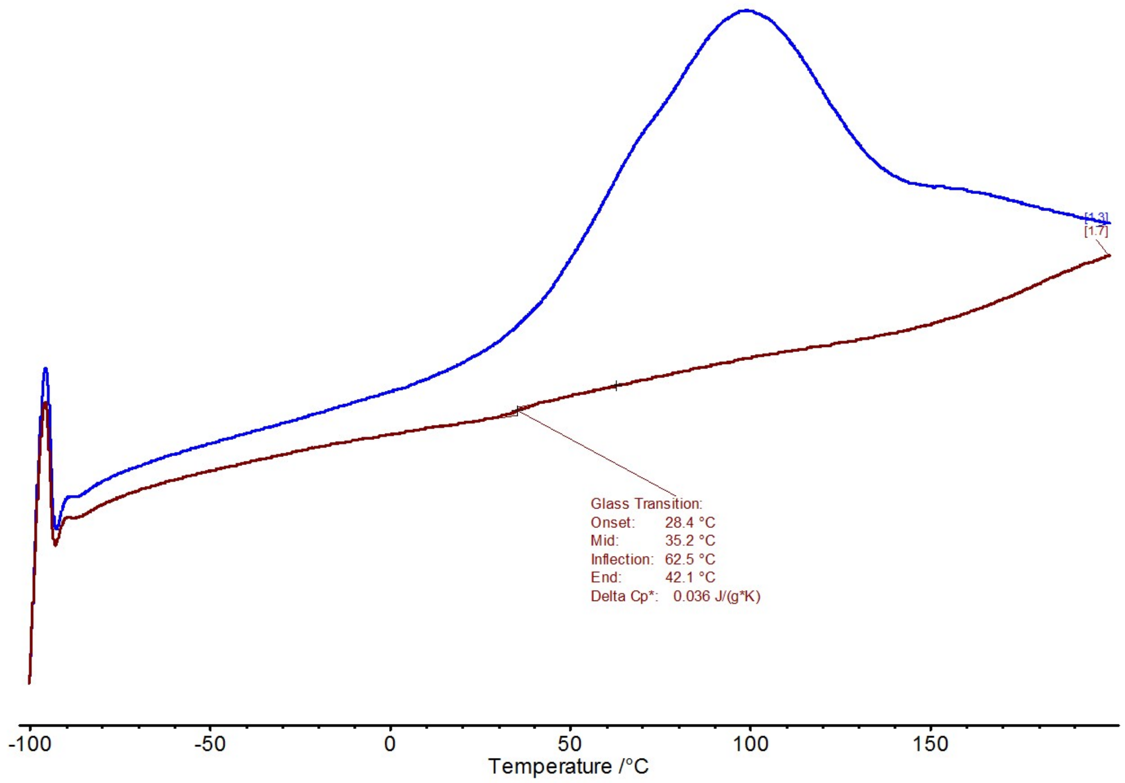

Figure S15. DSC of 1-allylimidazolium functionalized LS. Traces 1.3 and 1.7 correspond to the second and third heatings. Plotted as “exo down”.

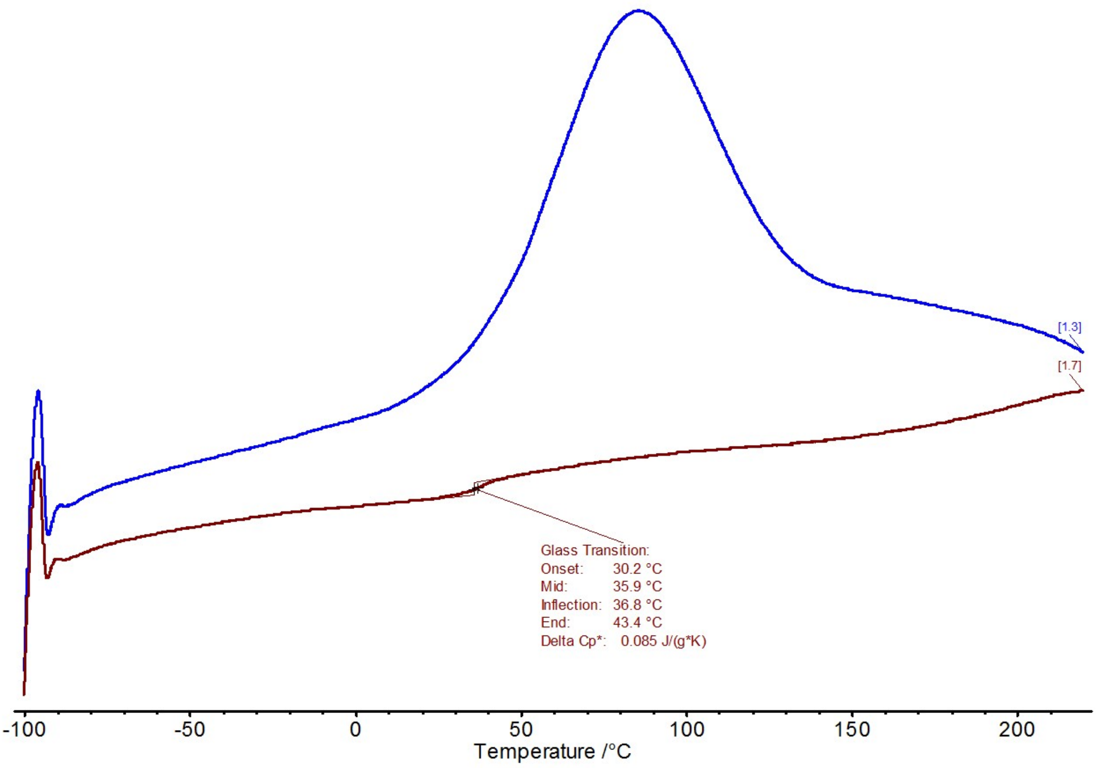

Figure S16. DSC of vinylimidazolium functionalized LS. Traces 1.3 and 1.7 correspond to the second and third heatings. Plotted as "exo down".

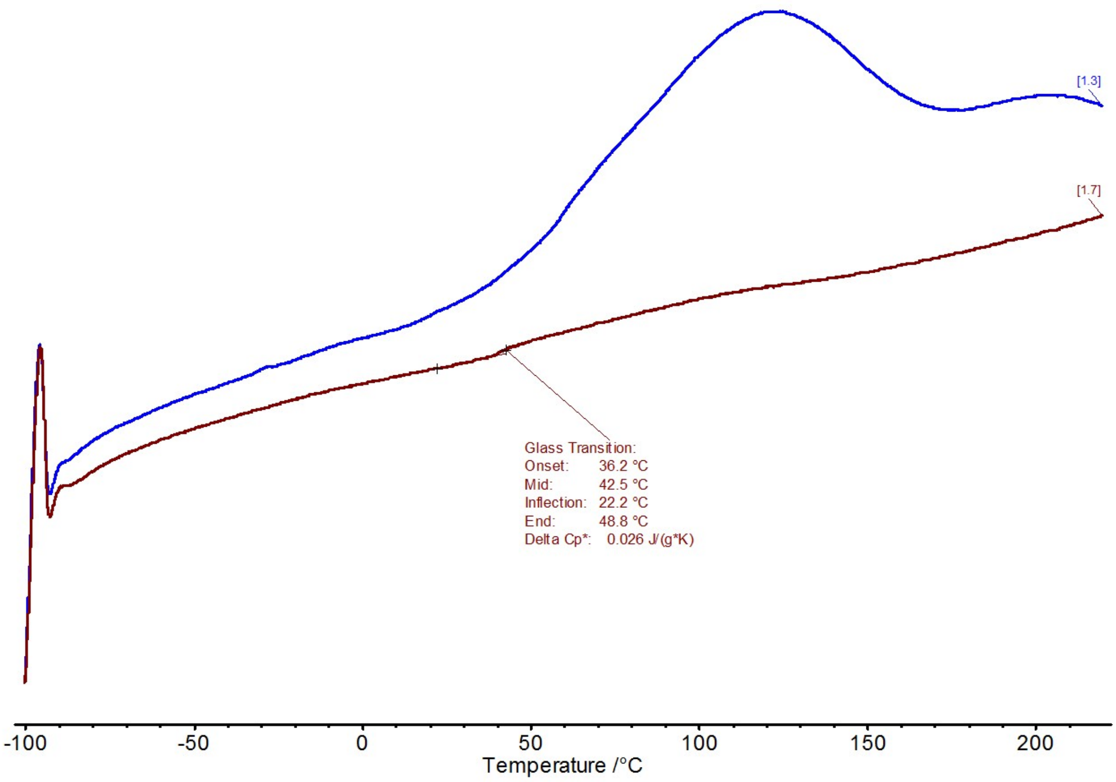

Figure S17. DSC of trioctylammonium functionalized LS. Traces 1.3 and 1.7 correspond to the second and third heatings. Plotted as "exo down".

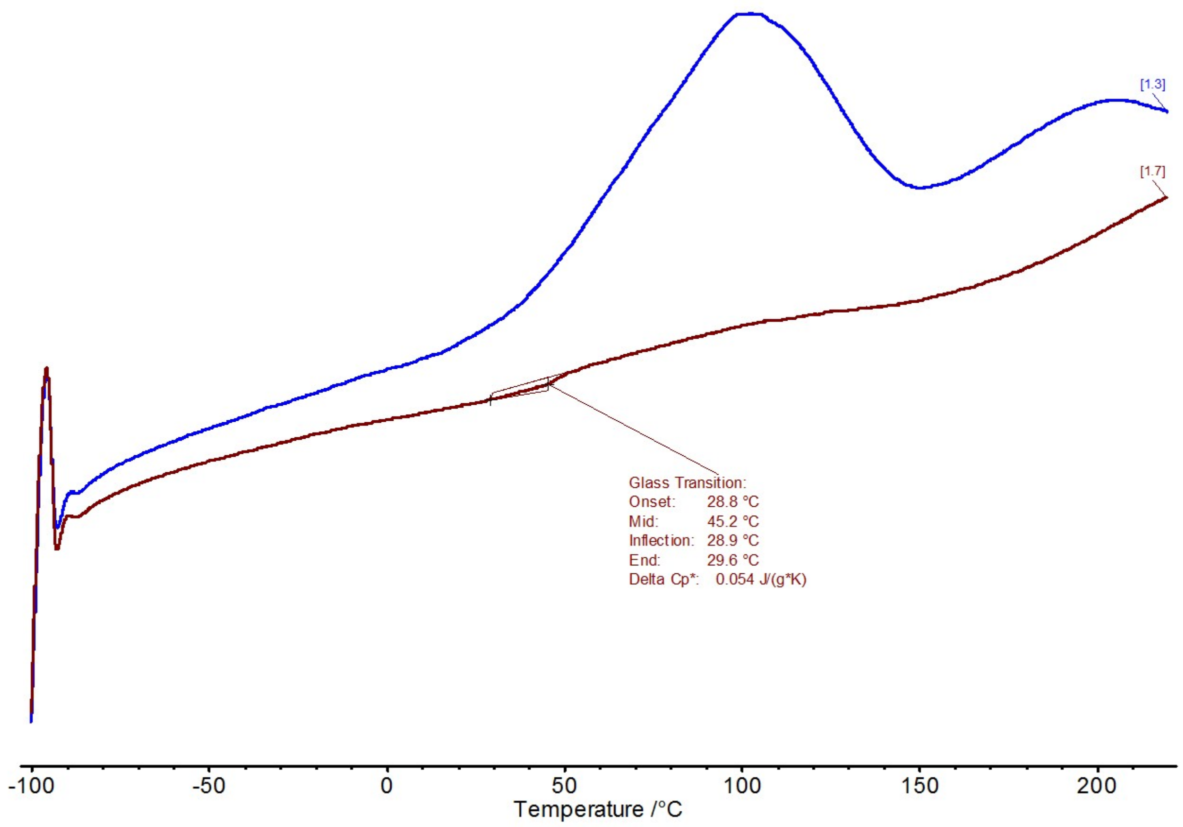

Figure S18. DSC of 1-methylimidazolium functionalized LS. Traces 1.3 and 1.7 correspond to the second and third heatings. Plotted as "exo down".

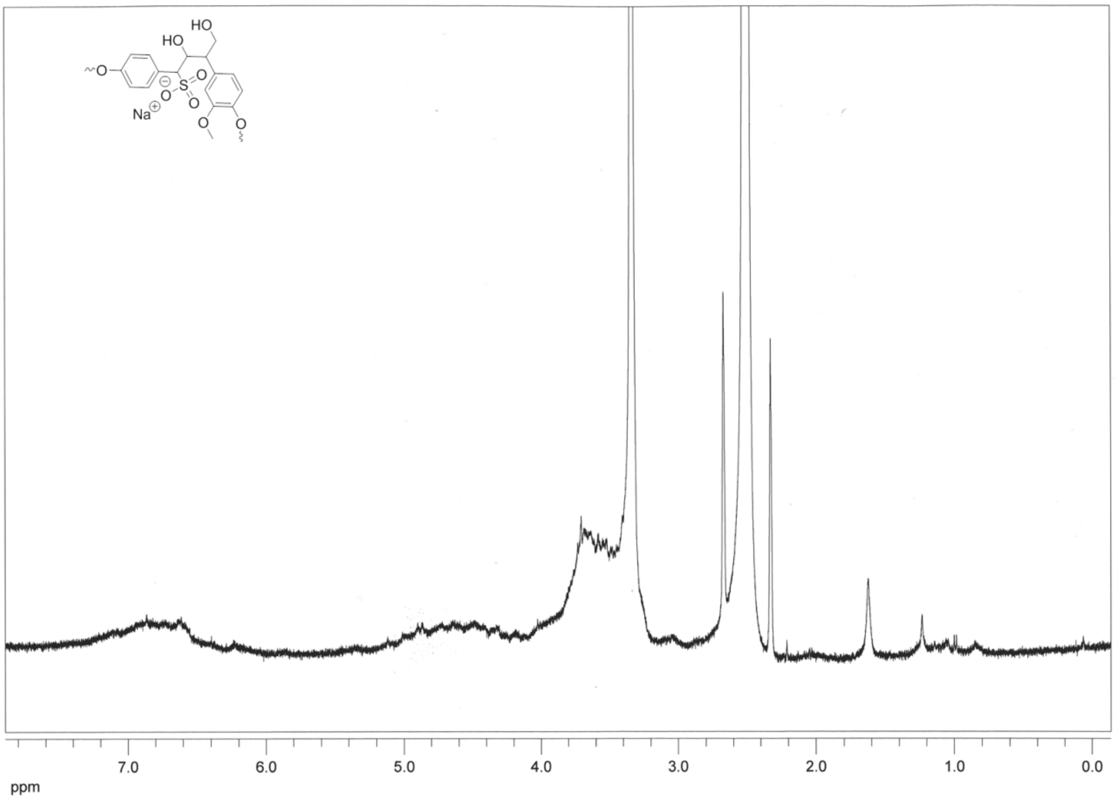

Figure S19. ^{1}H-NMR spectrum of commercial SLS in deuterated DMSO.

Table S3. Signal assignments for SLS in DMSO

**Signal (ppm)**	**Assignment**
7.20-6.80	Guaiacyl unit protons
6.80-6.20	Syringyl unit protons
5.00-4.00	H*α* in β-O-4’ structures, Hβ in β-O-4’ structures, H*γ* in β-β’ structures and H in xylan residues
4.00-2.90	H in methoxyls and Hβ in β-β’ structures
2.65-2.20	DMSO
1.65-0.80	Protons in aromatic and aliphatic acetates

See reference for further details on assignments of lignosuldonate in DMSO: Zhou, H.; Yang, D.; Zhu, J. Y. *J. Dispers. Sci. Technol.* **2016**, *37* (2), 296–303.

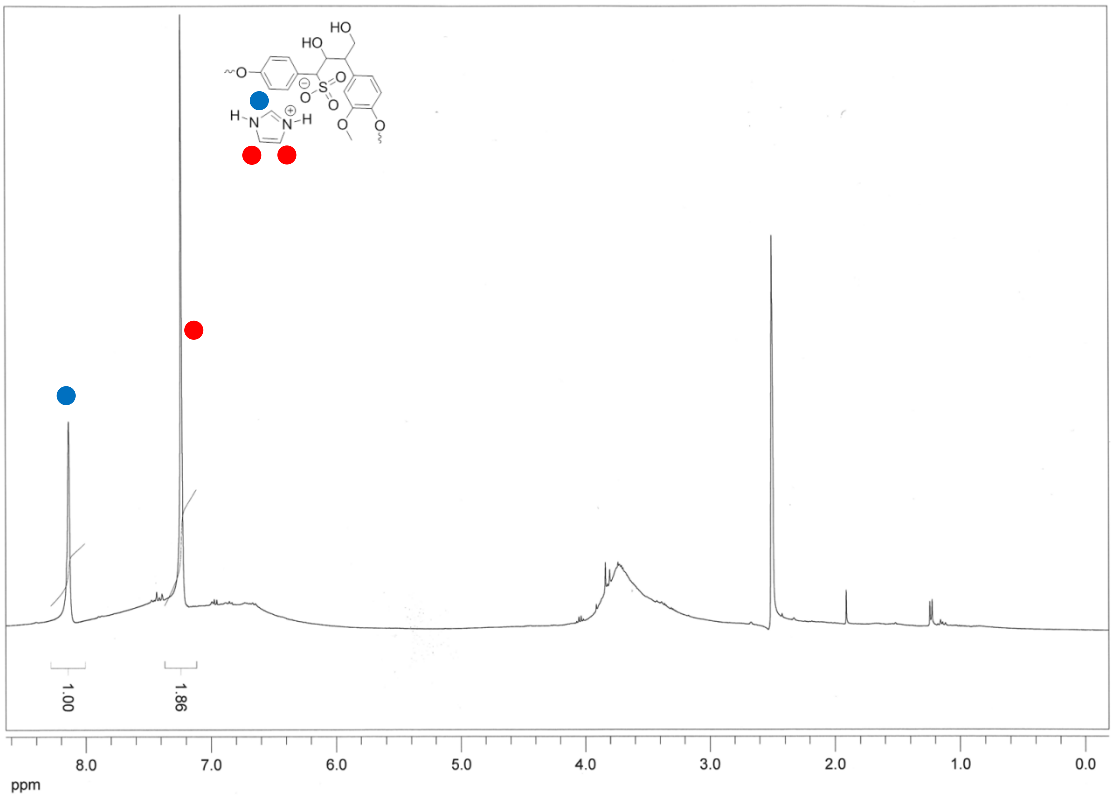

Figure S20. ^{1}H-NMR spectrum of imidazolium exchanged lignosulfonate in deuterated DMSO.

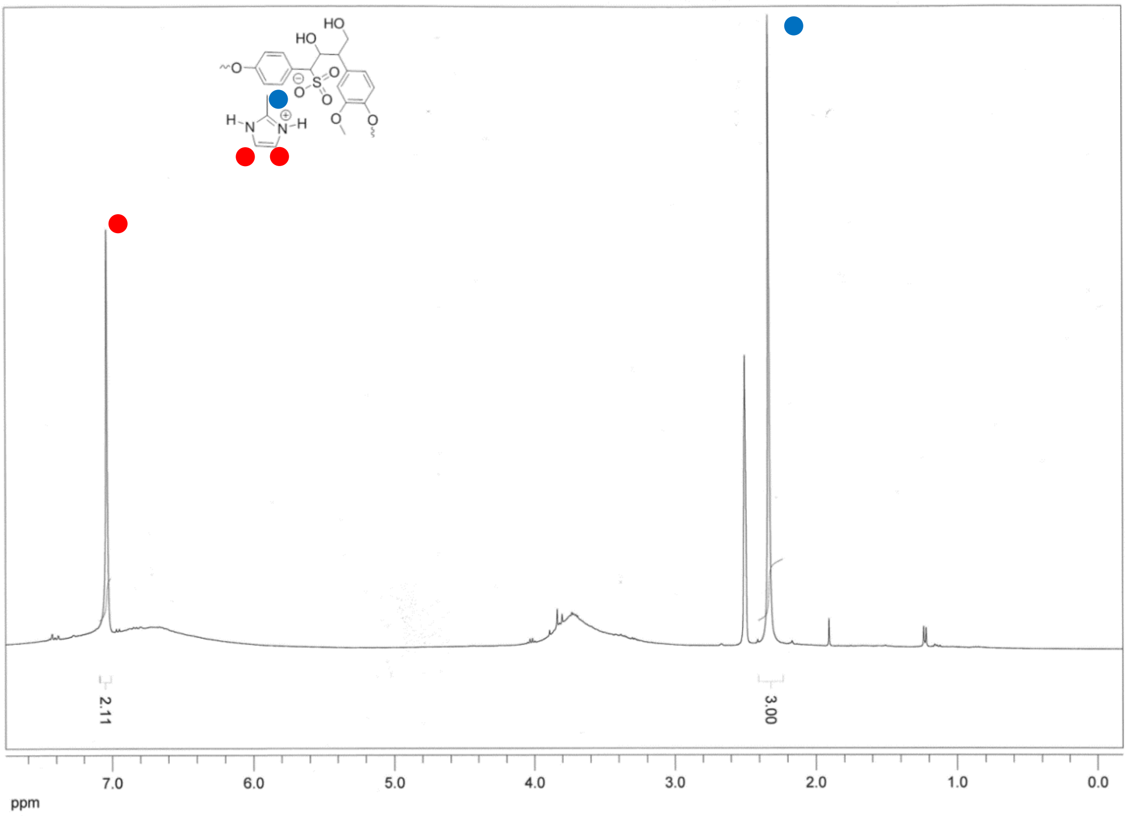

Figure S21. ^{1}H-NMR spectrum of 2-methylimidazolium exchanged lignosulfonate in deuterated DMSO.

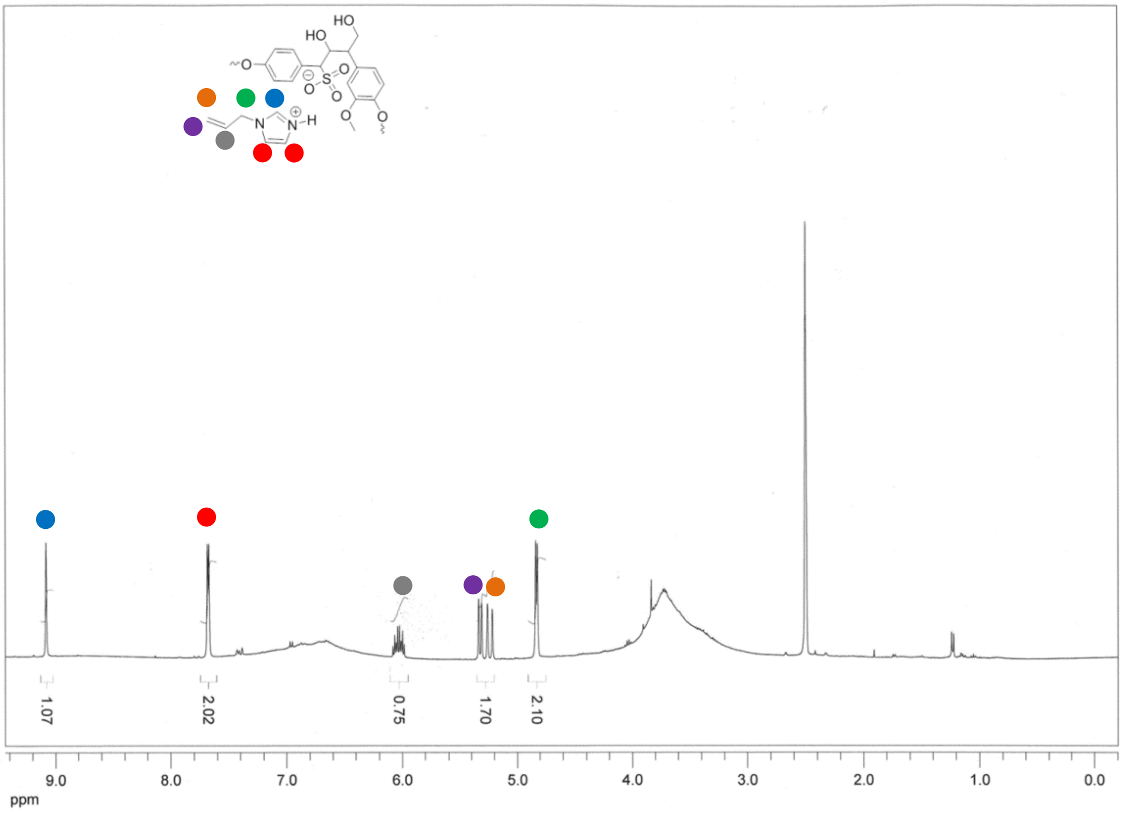

Figure S16. ^{1}H-NMR spectrum of 1-allylimidazolium exchanged lignosulfonate in deuterated DMSO.

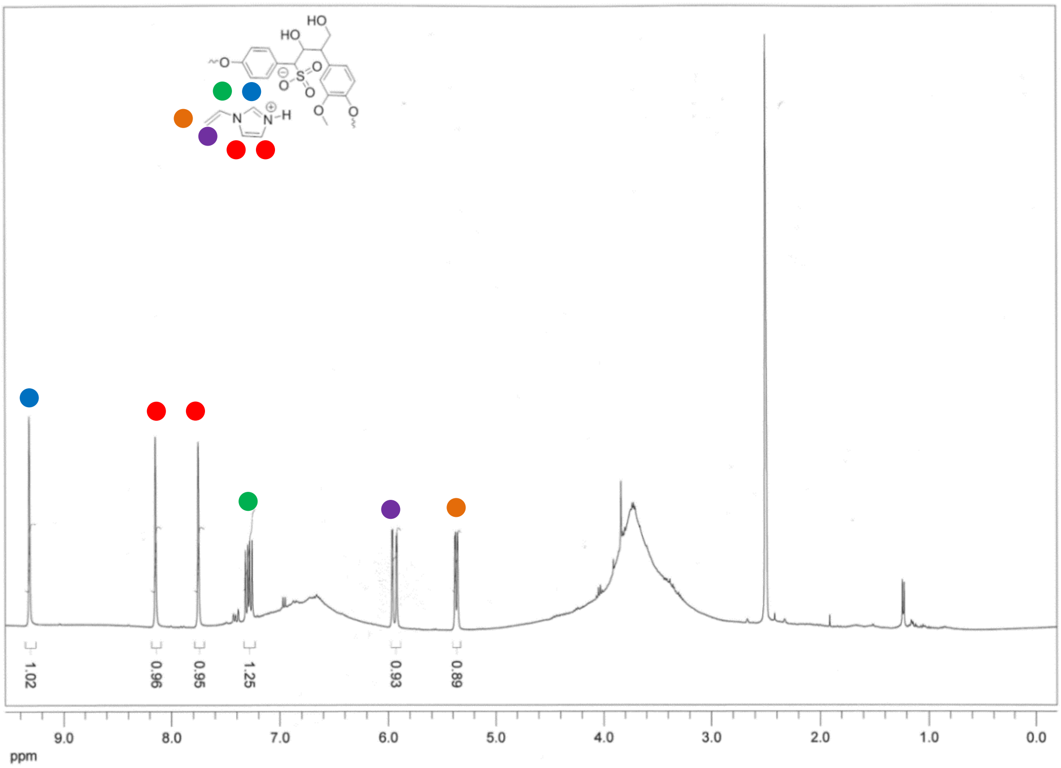

Figure S22. ^{1}H-NMR spectrum of 1-vinylmidazolium exchanged lignosulfonate in deuterated DMSO.

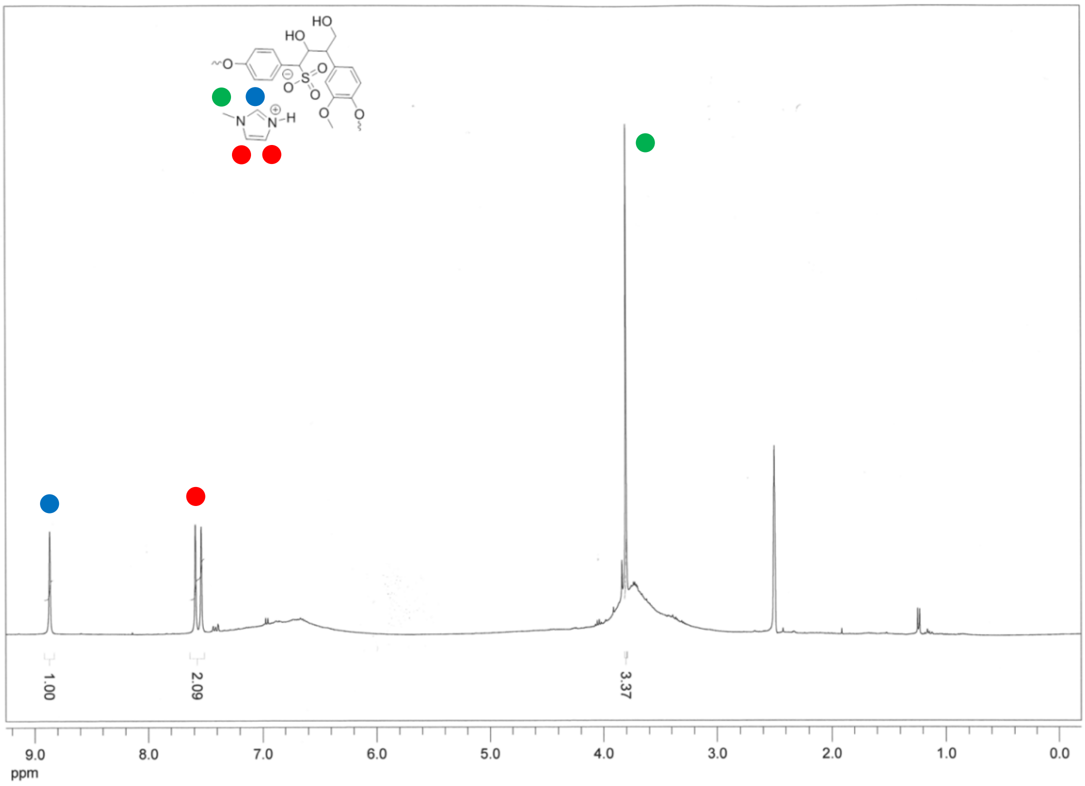

Figure S23. ^{1}H-NMR spectrum of 1-methylimidazolium exchanged lignosulfonate in deuterated DMSO.

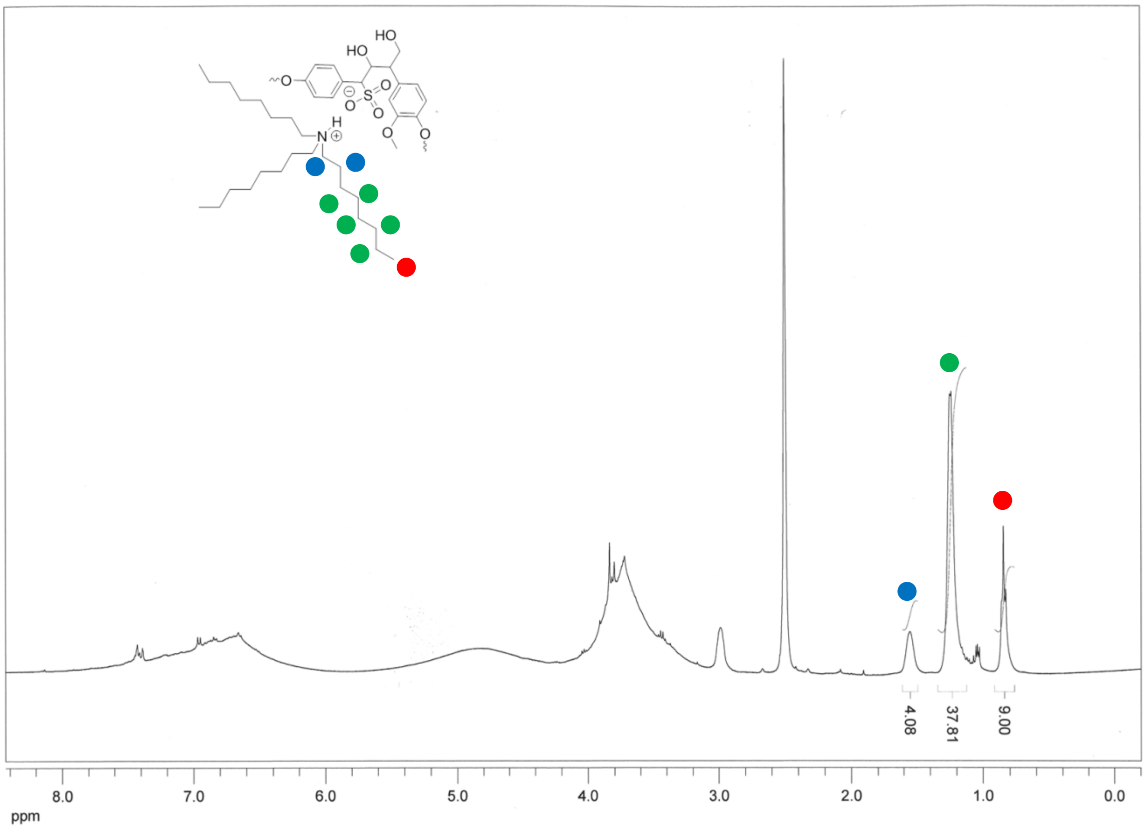

Figure S24. ^{1}H-NMR spectrum of trioctylammonium exchanged lignosulfonate in deuterated DMSO.

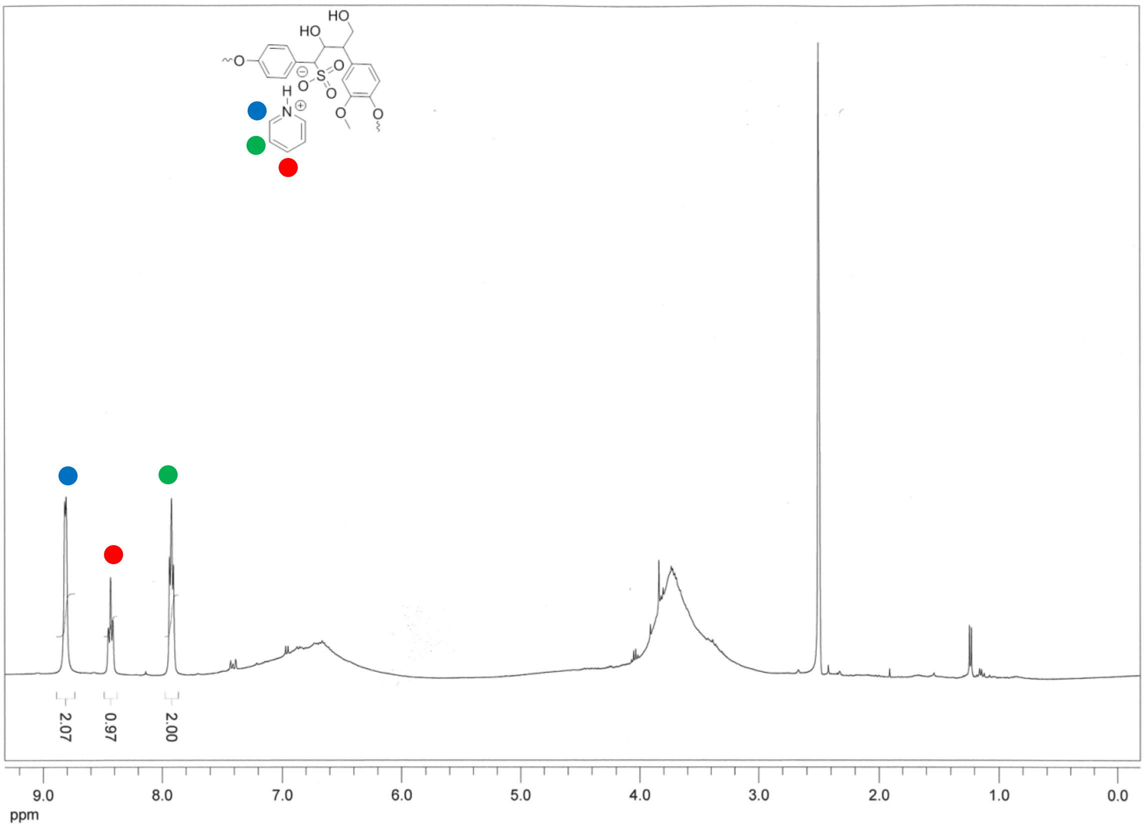

Figure S25. ^{1}H-NMR spectrum of pyridinium exchanged lignosulfonate in deuterated DMSO.

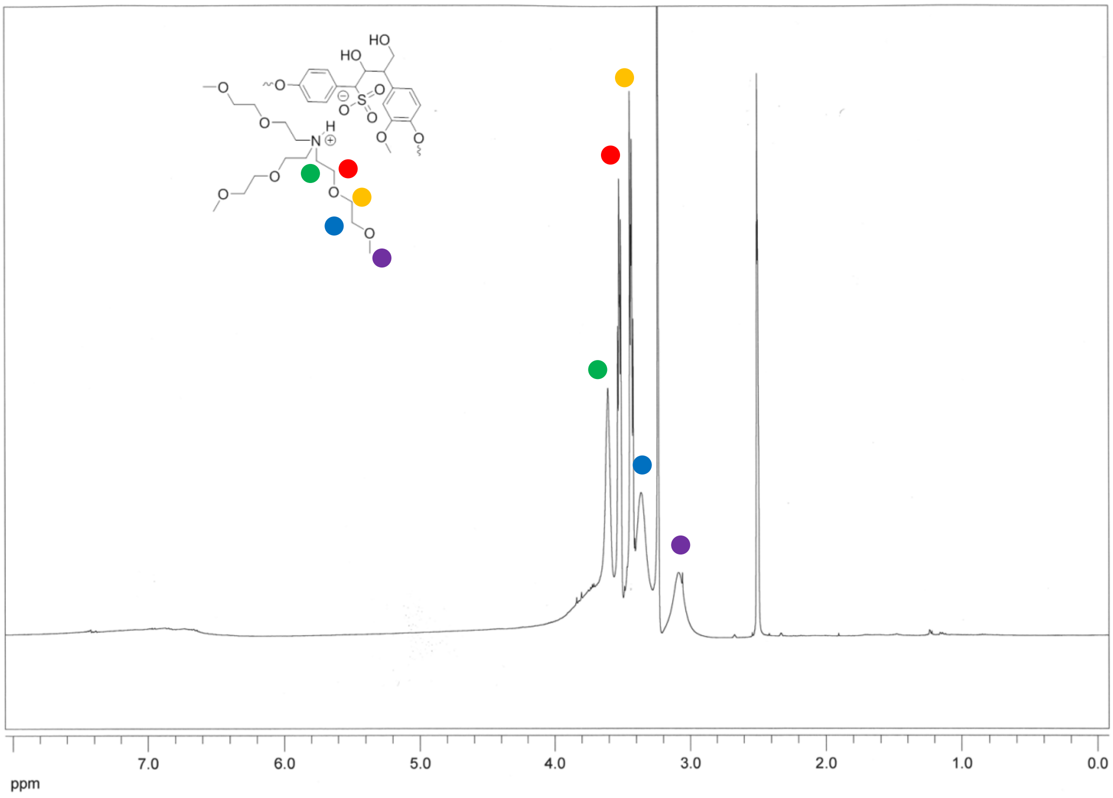

Figure S26. ^{1}H-NMR spectrum of **TrisEG** exchanged lignosulfonate in deuterated DMSO.

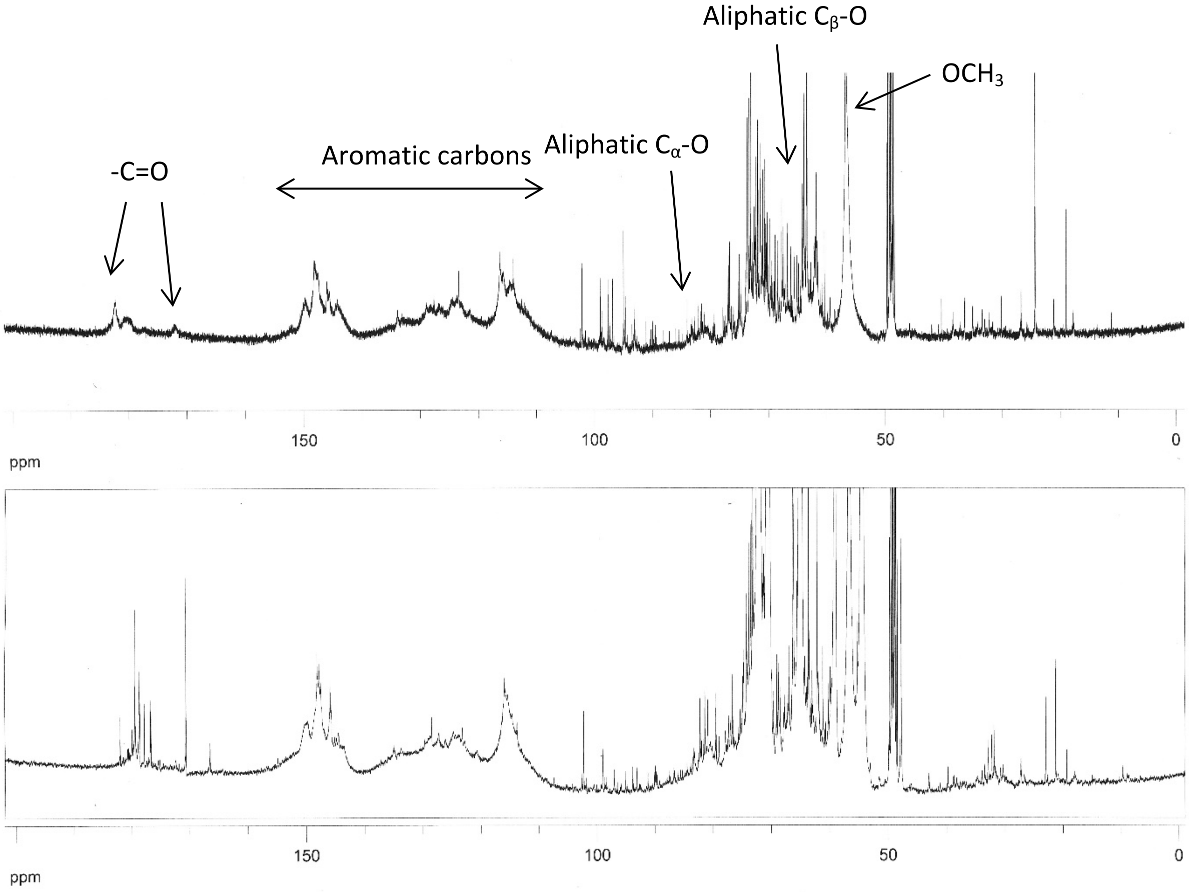

Figure S27. $^{13}C\{^1H\}$-NMR spectra of (top) **SLS** and (bottom) **TrisEG:LS** in D_2O spiked with deuterated methanol for referencing. The **SLS** used is likely a complex mixture of lignin and thus provides a complicated spectrum, however comparing the two samples there appears to be little difference in the peak positions of the aromatic and carbonyl regions. (Lu, Y.; Lu, Y.-C.; Hu, H.-Q.; Xie, F.-J.; Wei, X.-Y.; Fan, X. *J. Spectrosc.* **2017**, *2017*, 1–15.)

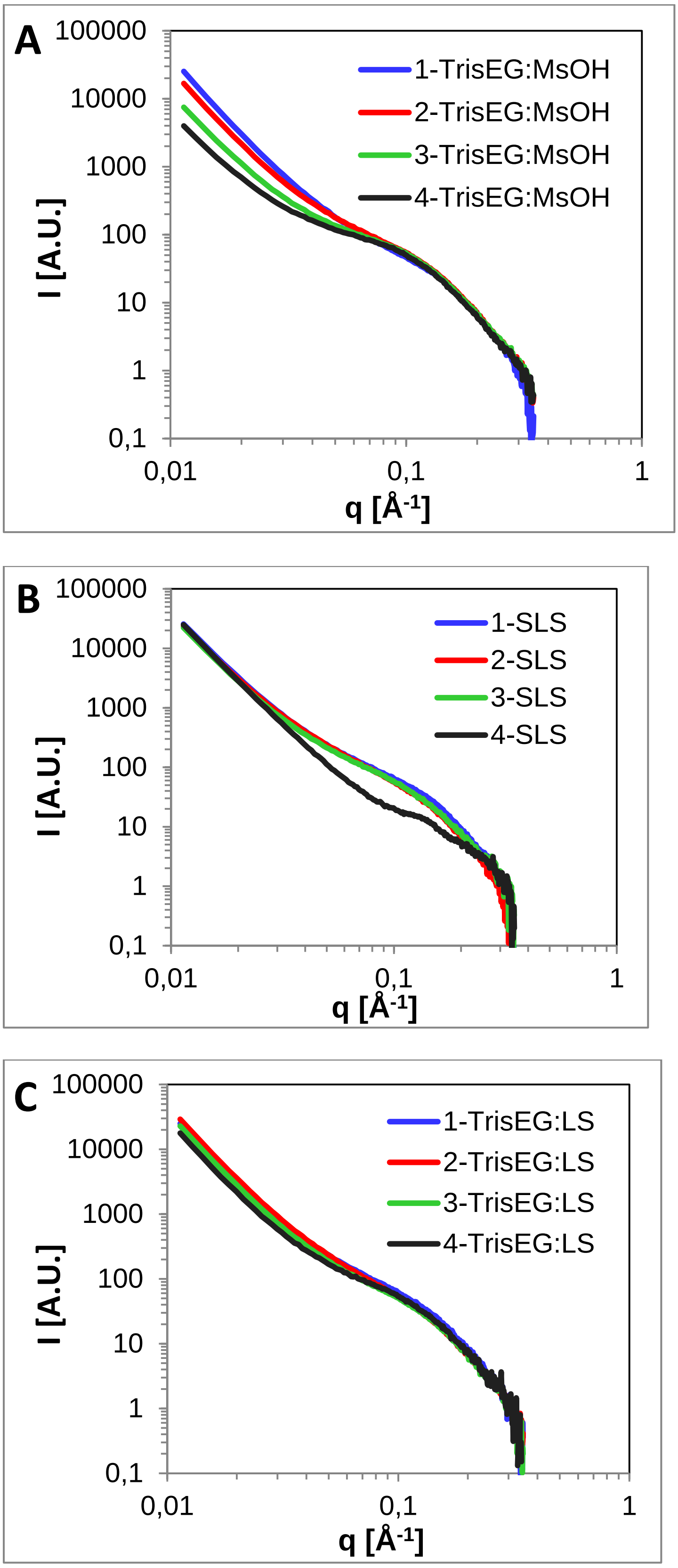

Figure S28. SAXS patterns of specimens with different concentrations of **TrisEG:MsOH** (A), **SLS** (B), and **TrisEG:LS** (C).

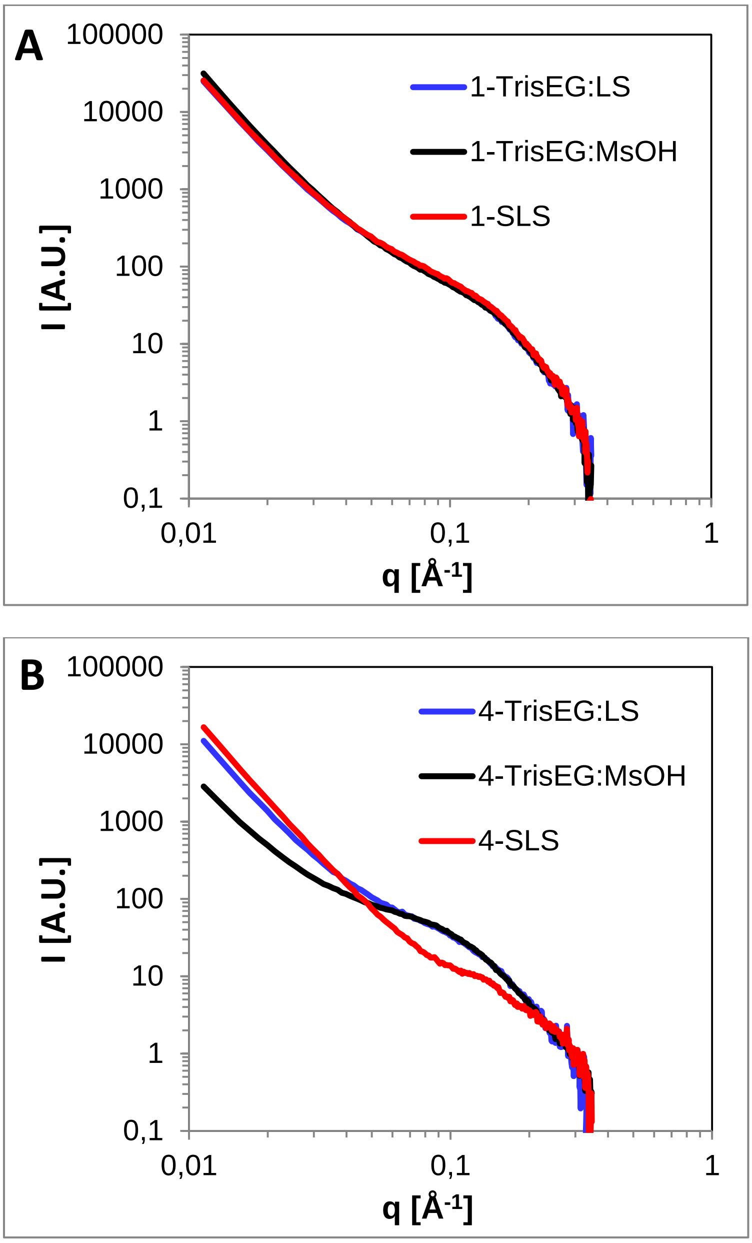

Figure S29. SAXS patterns of specimens with different dispersants **TrisEG:LS**, **TrisEG:MeOH**, and **SLS** at a concentration of 6 wt% (A) and 38 wt% (B).

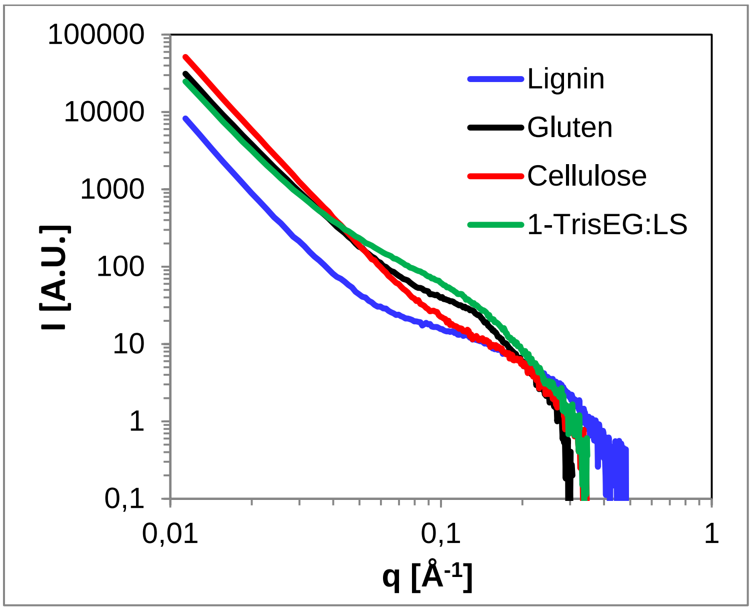

Figure S30. SAXS patterns of the unprocessed components lignin, gluten, and cellulose. **1-TrisEG:LS** appears for comparison. The scattering from unprocessed **TrisEG:MsOH** and **TrisEG:LS** is negligible.
